# Development and In Vitro Efficacy of Macrophage-Targeted Nanoparticles Encapsulating Vitamin E Derivatives Garcinoic Acid and α‑13′-Carboxychromanol

**DOI:** 10.1021/acsomega.6c01032

**Published:** 2026-08-21

**Authors:** Deniz Gol, Eda Ilayda Talaz, Seher Mese-Tayfur, Merve Acikel Elmas, Antimo Gioiello, Francesco Galli, Nesrin Kartal Ozer, Erdi Sozen, Ozgul Gok

**Affiliations:** † Department of Biomedical Engineering, Graduate School of Natural and Applied Sciences, Acibadem Mehmet Ali Aydinlar University, Istanbul 34752, Turkey; ‡ Department of Biochemistry, Faculty of Medicine, 52982 Marmara University, Istanbul 34854, Turkey; § Institute of Health Sciences, 52982 Marmara University, Istanbul 34854, Turkey; ∥ Department of Histology and Embryology, School of Medicine, Acıbadem Mehmet Ali Aydınlar University, Istanbul 34752, Turkey; ⊥ Laboratory of Medicinal and Advanced Synthetic Chemistry (Lab MASC), Department of Pharmaceutical Sciences, University of Perugia, Perugia 06122, Italy; # Human Nutrition and Nutrigenomics Lab, Department of Pharmaceutical Sciences, University of Perugia, Perugia 06122, Italy; ¶ Department of Biochemistry, Faculty of Medicine, 232990 Uskudar University, Istanbul 34662, Turkey; ∇ Metabolic and Inflammatory Diseases Research Center (METIFLAM), 232990 Uskudar University, Istanbul 34662, Turkey; ○ Genetic and Metabolic Diseases Research and Investigation Center (GEMHAM), 52982 Marmara University, Istanbul 34854, Turkey; ⧫ Department of Biomedical Engineering, Faculty of Engineering and Natural Sciences, Acibadem Mehmet Ali Aydinlar University, Istanbul 34752, Turkey

## Abstract

Garcinoic acid (GA), a phytomolecule and long-chain metabolite of δ-tocotrienol, and its analogue α-13′-carboxychromanol (α-13′-COOH), derived from α-tocopherol metabolism, are bioactive compounds involved in the regulation of various processes, including immune function and inflammatory responses. Pharmacological properties of these compounds are constrained by the rapid metabolism and the poor target selectivity in distinct tissue or cell types, including macrophages, a main player in the inflammatory response. To overcome these limitations, in this study, the DSPE-PEG-S2P conjugate containing a stabilin-2 receptor-specific S2P peptide was synthesized and utilized for the preparation of nanoparticles (NPs) for macrophage targeting. Also, GA or α-13′-COOH were loaded into nontargeted or macrophage-targeted NPs, and their delivery efficiency was compared against bone marrow-derived macrophages (BMDMs). These NPs exhibited stability for at least 2 days at room and body temperature and provided pH-responsive drug release. Moreover, macrophage-targeted NPs demonstrated a linear dose-dependent increase of cellular levels for both metabolites, whereas nontargeted NPs induced dose-independent accumulation, which emphasizes the targeting ability of the S2P peptide. Additionally, both NPs had no adverse effect on cell morphology and viability. Present findings confirmed the improving effect of S2P-decorated NPs for delivering GA or α-13′-COOH metabolites specifically into these inflammatory cells via targeting macrophages. Cell selectivity and efficacy of this delivery system deserve further preclinical investigation in models of acute and chronic inflammation.

## Graphical abstract

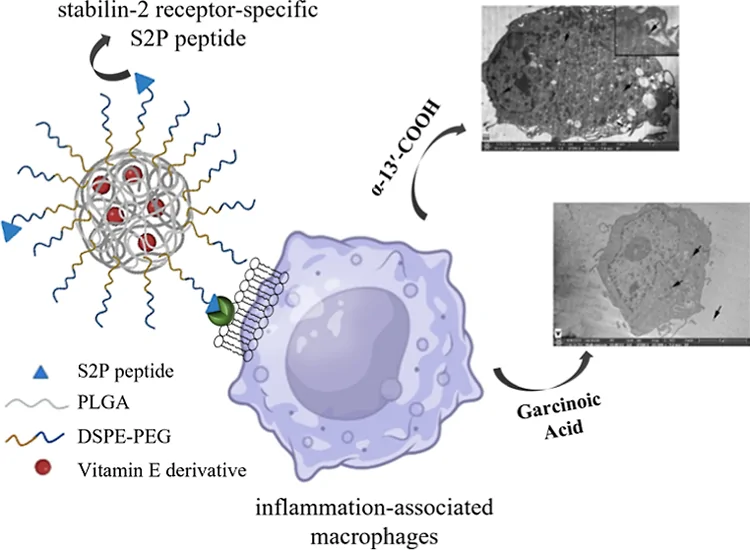

## 1 Introduction

Since its discovery by Evans and Bishop in 1922, vitamin E and its analogues (α-, β-, γ-, δ-tocotrienol and α-, β-, γ-, δ-tocopherol) have been shown to be effective in modulating signaling pathways and disease development.^1,2^ However, the identification of metabolites that are produced during the hepatic metabolism of vitamin E opens a novel area of interest. Studies have classified the biological activities of these metabolites into four groups: (i) anti-inflammatory effect, (ii) anticarcinogenic effect, (iii) modulation of cellular lipid homeostasis, and (iv) interaction with pharmaceutical compounds.^3–5^ Garcinoic acid (GA or *trans*-13′-carboxy-δ-tocotrienol) is a natural δ-tocotrienol derivative that is extracted from *Garcinia kola*, while α-13′-carboxychromanol (α-13′-COOH) is produced during the hepatic metabolism of α-tocopherol. Despite studies revealing their role in in vitro and in vivo experimental models, their rapid metabolism and, therefore, low concentrations in plasma limit their therapeutic applications.^6,7^

Efficient delivery of these metabolites using nanosized particles made out of natural or synthetic biomaterials has become a major role in biomedical research.^8^ Nanoparticles (NPs) stand out as promising carrier systems with their ability to protect the therapeutics from degradation in the gastrointestinal tract, to remain in the systemic circulation for a longer period, to enable the delivery to the target area, and to provide their controlled release.^9,10^ The literature covers various NP types that help increase the bioavailability of drugs compared to their free forms.^11,12^ Especially PEG (poly­(ethylene glycol)) attachment to the surface of nanoparticles, known as PEGylation, has been shown to improve the surface hydrophilicity and exhibit reduced protein binding and partial preservation of targeting capability in biological environments.^13^ Additionally, certain studies use NP-based carriers to specifically target and deliver bioactive substances to the target site depending on specific ligand–receptor interactions.^14–16^ Among diseases dependent on inflammation like atherosclerosis, NPs have been shown to be directed to disease-associated macrophages in the blood.^17^ The discovery of the S2P peptide that recognizes the macrophage receptor Stabilin-2 is a promising development in the fight against inflammatory diseases, encouraging further investigation of their medical applications.^18,19^ In 2020, Tao and co-workers reported the successful decoration of PLGA-based siRNA NPs with the S2P peptide, which helps accumulation of NPs in macrophages, utilizing high-affinity biochemical bonds to anchor to the target surface even under flow conditions, thereby inhibiting atherosclerotic plaque necrosis.^19^

In light of these findings, the present study aims to utilize a very promising strategy to deliver vitamin E derivatives, namely, GA and α-13′-COOH, to inflammation-associated macrophages that overexpress the Stabilin-2 receptor. This study describes a one-step method for preparing PLGA-based NPs encapsulating either GA or α-13′-COOH, together with the S2P peptide on their surface for better targeting ability. By using bone marrow-derived macrophages (BMDMs), the efficiency of these NPs was evaluated in in vitro conditions, and their increased accumulation was successfully demonstrated for macrophage-targeted ones. The results indicate that S2P-conjugated NPs improve delivery of GA and α-13′-COOH to inflammatory cells, supporting their potential as effective carriers for preclinical studies.

## 2 Materials and Methods

### 2.1 Chemicals and Supplies

Poly­(lactic-*co*-glycolic acid) (PLGA, cat no: 805726), *N*,*N*-dimethylformamide (DMF, cat no: 227056), triethylamine (Et_3_N, cat no: T0886), acetic acid (cat no: 27225-M), triisopropylsilane (TIS, cat no: 233781), Fmoc-Arg­(Pbf)-OH Novabiochem (arginine, cat no: 8.52067), Fmoc-Cys­(Trt)-OH Novabiochem (cysteine, cat no: 8.52008), Fmoc-Lys­(Boc)-OH Novabiochem (lysine, cat no: 8.52012), Fmoc-Thr­(*t*Bu)-OH Novabiochem (threonine, cat no: 8.52000), Fmoc-Val-OH Novabiochem (valine, cat no: 8.52021), and Oxyma pure Novabiochem (cat no: 8.51086) were purchased from Sigma-Aldrich (MA, USA). 1,2-Distearoyl-*sn*-glycero-3-phosphoethanolamine-*N*-[methoxy­(polyethylene glycol)-3000] (DSPE-PEG, cat no: 880320C) was acquired from Avanti Polar Lipids (AL, USA). DSPE-PEG-Maleimide (cat no: PG2-DSML-3k) was purchased from Nanocs Inc. (NY, USA). Piperidine (cat no: 109724), *N*,*N*′-diisopropylcarbodiimide (DIC, cat no: 803649), *N*,*N*-dimethylformamide for peptide synthesis (DMF, cat no: 100397), diethyl ether (cat no: 1.00921), and trifluoroacetic acid (TFA, cat no: 108178) were acquired from Merck (NJ, USA). Acetonitrile (cat no: 901.037) was purchased from ISOLAB Chemicals (Laborgeräte GmbH, Wertheim, Germany). Fmoc-l-Leu-OH (leucine, cat no: A011-C) and Rink Amide proTide (resin, cat no: R003-C) were obtained from CEM Corporation (NC, USA). The Spectra/Por 6 2000 D MWCO standard RC prewetted dialysis kit (cat no: 08-700-193, Spectrum Laboratories) and phosphate-buffered saline (PBS) tablets (cat no: 2810305) were obtained from Thermo Fisher Scientific and MP Biomedicals, respectively (CA, USA). Dulbecco’s Modified Eagle’s Medium (DMEM, cat no: 21969-035), fetal bovine serum (FBS; cat no: 10108-165), l-glutamine (cat no: 25030-024), and penicillin–streptomycin mixture (cat no: 15140-122) were acquired from Gibco, Invitrogen. α-13′-COOH and GA were obtained as previously reported.^20^

### 2.2 Nontargeted Nanoparticle Synthesis

Nontargeted NPs were synthesized as previously described in the literature, with minor parameters adjusted according to optimization experiments.^21^ To synthesize nontargeted empty NPs, PLGA (2.5 mg, 0.05 μmol) was dissolved in 500 μL of acetone, while DSPE-PEG (2 mg, 0.53 μmol) was dissolved in 10 mL of ddH_2_O. The PLGA solution was added dropwise to the DSPE-PEG solution, and the mixture was stirred for 1 h at 1100 rpm. After stirring for 1 h at room temperature (RT) in an open-top beaker to allow the evaporation of acetone, the NP solution was then centrifuged three times against ddH_2_O for 20 min at 1649 × *g* to remove impurities. The purified nontargeted NPs were resuspended in 1 mL of ddH_2_O and stored at −20 °C for future use. The same procedure was followed for the synthesis of drug-loaded nontargeted NPs, with the exception that either α-13′-COOH (0.25 mg, 0.54 μmol) or GA (0.25 mg, 0.59 μmol) was dissolved in 500 μL of acetone together with PLGA (2.5 mg, 0.05 μmol). After the synthesis, the drug-loaded NPs were centrifuged to remove unbound drug molecules and impurities as previously described. Finally, the purified NPs were resuspended in 1 mL of ddH_2_O and stored at −20 °C.

### 2.3 S2P Peptide and DSPE-PEG-S2P Conjugate Synthesis

The stabilin-2 receptor-specific S2P peptide (CRTLTVRKC) was synthesized using the Liberty Blue Automated Microwave Peptide Synthesizer (CEM), following a previously reported method.^22^ The synthesis scale was adjusted to 0.1 mmol, with 0.143 g of resin swollen for at least 30 min before starting the peptide synthesis. For the peptide synthesis, amino acid solutions were prepared in DMF. DIC and Oxyma were used as an activator and activator base, respectively, while piperidine was used for deprotection by mixing 1.1 mL of DIC with 12.9 mL of DMF, and the piperidine solution was obtained by adding 40 mL of DMF to 10 mL of piperidine. Separately, 0.995 g of Oxyma was dissolved in 7 mL of DMF. At the end of the synthesis, the peptide was cleaved from the resin with the RazorTM Peptide Cleavage System (CEM) at 37 °C for 30 min. The cleavage cocktail consists of 4.75 mL of TFA, 125 μL of TIS, and 125 μL of ddH_2_O. Then, the obtained S2P peptide was precipitated using cold diethyl ether and centrifuged at 6000 rpm for 3 min against cold diethyl ether three times. The pellet was dried in the desiccator and then purified using a preparative High-Performance Liquid Chromatography (prep-HPLC, The CombiFlash NextGen 300+, EZ Prep) device to remove impurities that may be present in the reaction mixture, using RediSep Silver C18 Reversed Phase columns. The chemical characterization of the synthesized S2P peptide was completed using an LC/MS–MS device (6420 Triple Quad LC/MS, Agilent Technologies). At the end, the peptide was lyophilized using a HyperCOOL 8080 instrument (LaboGene A/S, Lynge, Denmark) and stored at 4 °C.

The DSPE-PEG-S2P conjugate was synthesized as previously reported.^23^ Briefly, the peptide (19 mg, 0.018 mmol, 1.2 equiv) was conjugated to DSPE-PEG-Maleimide (50 mg, 0.015 mmol, 1 equiv), which has a free maleimide group at one side, using a 1,4 Michael addition reaction (Figure 1). After lyophilization, the final product was purified using a dialysis membrane (Mwt cutoff: 2 kDa) and stored at 4 °C. The chemical structure of the synthesized conjugate was confirmed by both proton nuclear magnetic resonance (^1^H NMR) (Bruker 400 MHz in DMSO-*d*_6_) and Fourier-transform infrared (FT-IR) spectroscopy (NICOLET iS10; Thermo Scientific).

**Figure 1:**
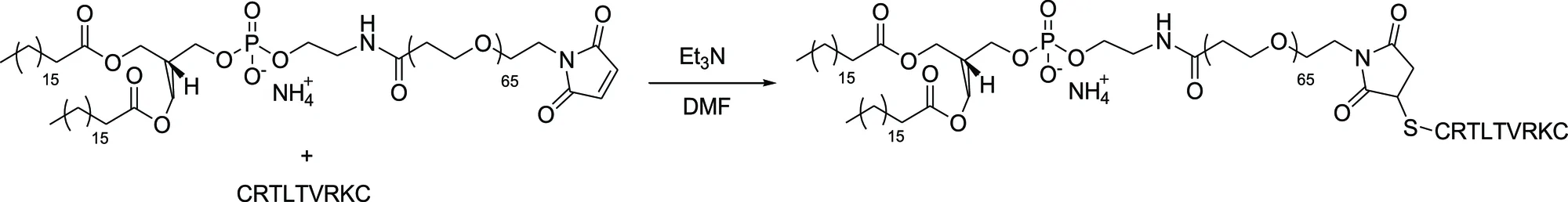
Synthesis of the DSPE-PEG-S2P conjugate.

### 2.4 Macrophage-Targeted Nanoparticle Synthesis

Macrophage-targeted NPs were synthesized by dissolving PLGA (2.5 mg, 0.05 μmol) in 500 μL of acetone. DSPE-PEG (1.8 mg, 0.48 μmol) and DSPE-PEG-S2P (0.2 mg, 0.044 μmol) were dissolved in 10 mL of ddH_2_O, and the PLGA solution was added dropwise to this mixture.^21^ The solution was stirred for 1 h at 1100 rpm in an open-top beaker. The NPs were then centrifuged three times with ddH_2_O for 20 min at 1649 × *g* to remove impurities. The purified, macrophage-targeted empty NPs were resuspended in 1 mL of ddH_2_O. For the synthesis of macrophage-targeted drug-loaded NPs, α-13′-COOH (0.25 mg, 0.54 μmol) or GA (0.25 mg, 0.59 μmol) was dissolved in 500 μL of acetone along with PLGA (2.5 mg, 0.05 μmol). The same steps were followed, and the prepared NPs were centrifuged to eliminate unbound drug molecules and impurities. The NPs were then resuspended in 1 mL of ddH_2_O, followed by the removal of aggregates, and stored at −20 °C.

### 2.5 Evaluation of Nanoparticle Characteristics

The comprehensive characterization of the synthesized nanoparticles (NPs) involved several analytical techniques to ascertain their physical properties, morphology, and targeting molecule content. The hydrodynamic diameter and the PDI of the NPs were assessed using a DynaPro NanoStar instrument (Wyatt Technologies, CA, USA). The procedure involved three repetitions for each sample, where every repetition consisted of 10 measurement runs following a 5 s equilibrium time at room temperature (RT). The surface charge of the NPs was determined using a Zetasizer Nano ZS (Malvern Panalytical, Malvern, UK) at RT. NPs were diluted to 10% by volume using a 10 mM NaCl solution, and zeta potential values were measured in triplicate, with each measurement encompassing 10 replicate runs. Fourier-transform infrared (FT-IR) spectroscopy was applied to confirm the presence of characteristic functional groups within the samples by comparing their spectral signatures against those of the precursor components. A Nicolet iS10 (Thermo Scientific) was used for data acquisition. Each sample spectrum was recorded at RT, covering the range from 400 to 4000 cm^–1^ with 32 scans. The morphology of the NPs was visualized using both Transmission Electron Microscopy (TEM) and Scanning Electron Microscopy (SEM). For TEM (performed with a Thermo Fisher Scientific, Talos L120C), 20 μL portions of the NP solutions were deposited onto copper TEM grids that were supported by a lacey carbon film. The samples were then allowed to air-dry for approximately 48 h in a controlled environment. Images were subsequently captured under conditions of low pressure and high vacuum. For SEM (performed with a Thermo Fisher Scientific Quattro ESEM), NP solutions were applied to carbon-taped metal stubs and air-dried in a sterile setting for 48 h. The samples were then subjected to gold coating under vacuum. SEM images were collected at 20 kV using a 3.5 spot size. The stability of the NPs was investigated by measuring their hydrodynamic volume via DLS, with three repetitions completed for predetermined time points at different pH environments. Size measurements were done upon incubation either in 10 mM PBS (pH 7.4) or acetate buffer (pH 5.5) at two different temperatures, RT and 37 °C. The net concentration of the S2P peptide present on the macrophage-targeted NPs was determined spectrophotometrically. A Thermo Scientific NanoDrop One Microvolume UV–vis Spectrophotometer was utilized for this purpose. Specifically, a standard curve was generated from eight serial dilutions of the peptide standard measured at 214 nm using a NanoDrop spectrophotometer (Thermo Scientific NanoDrop One) (Figure S9). A 2 μL aliquot of the NP solution was deposited onto the pedestal, and the final peptide concentration was calculated based on the average of measurements performed in triplicate for each sample.

### 2.6 Drug Encapsulation Efficiency and Drug Release Profile of Nanoparticles

The amount of drug molecules (α-13′-COOH or garcinoic acid) encapsulated in nanoparticles (NPs) was quantified utilizing an LC–MS/MS instrument (6420 Triple Quad LC/MS, Agilent Technologies). After the purification of NPs from any impurities and unencapsulated drug molecules via an Amicon tube-based filtration procedure, the obtained NP solutions were lysed by mixing them with acetone in a 1:1 volume ratio, leading to the dissociation of polymer chains and the liberation of the encapsulated drug molecules into the environment. The concentration of the released drug was subsequently determined by referencing pre-established calibration curves created for either α-13′-COOH or GA. A C18 column (10 cm × 2.0 mm, 3.0 μm particle size) was utilized as the stationary phase for liquid chromatography analysis, and an equi-volume mixture of acetonitrile and distilled water with 0.5% formic acid (v/v) was passed through the system as the mobile phase with a flow rate of 0.5 mL/min. Dynamic multiple reaction monitoring (MRM) methods were generated for both drug molecules separately, where the *m*/*z* value was determined as 459.2 for α-13′-COOH (Mwt_theo_: 460.7 g/mol) and as 364.9 for GA (Mwt_theo_: 426.6 g/mol) molecules. The drug encapsulation efficiency (DEE, %) values for each drug molecule were calculated by the ratio of the measured drug amount to the loaded amount.

Drug release profiles for both nanoparticles were assessed by the dialysis method, where a dialysis tube with a Mwt cutoff value of 2 kDa was filled with 1 mL of drug-loaded NPs. The membranes were immersed in 40 mL of receptor solutions and incubated at 37 °C. The incubation process was carried out in an orbital shaker (Ohaus Incubating Light Duty Orbital Shaker) set to maintain agitation at 200 rpm. To simulate relevant biological environments, two distinct release media were employed for assessing the drug release characteristics of the NPs: a pH 7.4 phosphate-buffered saline (PBS) solution, which mimics healthy physiological fluids, and a pH 5.5 acetate buffer solution, which represents the acidic conditions typical of inflammatory states. At different time points, samples were withdrawn from these release media, followed by the addition of a fresh one with the same volume. The released drug molecules in these samples were analyzed quantitatively using the LC–MS/MS instrument. The obtained quantitative release data were processed by fitting them to the Korsmeyer–Peppas mathematical model to interpret the drug release kinetics. The model uses eq 1.^24–26^

1 $$\left(\frac{Mt}{M\infty }\right)=Kt^{n}$$

### 2.7 Cell Culture and Primary Cell Isolation

L-929 cells (mouse fibroblasts) were grown in DMEM supplemented with 10% (v/v) heat-inactivated FBS, 1% penicillin–streptomycin, and 2 mM glutamine. Cells were maintained at 37 °C and 5% CO_2_ with a water reservoir for humidity control.^27^ To obtain an L-929 conditioned medium, the media were collected every week, stored at −80 °C, and used within 2 months.

Bone-marrow cells were isolated and differentiated into BMDMs according to a previous study.^28^ Briefly, bone marrow was collected from 8–12 week-old mice, and cells were cultured in DMEM supplemented with 10% (v/v) heat-inactivated FBS, 1% penicillin–streptomycin, and 20% (v/v) L-929 fibroblast-cultured medium supernatants. Every three days, cells were washed twice with Dulbecco’s phosphate-buffered saline (DPBS) and a fresh medium was added. After 7–9 days, cells were treated as described in the figure legends. Acibadem Mehmet Ali Aydinlar University Ethics Committee guidelines were followed in isolating mouse BMDMs (Approval number: 2021/53).

### 2.8 MTT Assay

The dose-dependent effects of GA and α-13′-COOH-loaded NPs on macrophage viability were evaluated by the 3-(4,5-dimethyl-thiazol-2-yl)-2,5-diphenyl-tetrazolium bromide (MTT) assay (Sigma-Aldrich). Cells were incubated with MTT for 2 h, and the absorbance was measured at 570 nm. Relative cell viability was calculated by comparison with untreated control cells.^20,29^

### 2.9 Transmission Electron Microscopy

BMDMs were fixed in 2.5% glutaraldehyde in PBS (0.1 M, pH 7.2) for 1 h and then postfixed with 1% osmium tetroxide in PBS (0.1 M, pH 7.2) for an additional hour at RT. The pellet was dehydrated with increasing concentrations of ethyl alcohol and embedded in Epon 812 resin. Ultrathin sections (600 A°) were stained with 2% uranyl acetate and lead citrate. Ultrathin sections were photographed and examined under a scanning electron microscope in STEM mode (Thermo Fisher Scientific-Quattro).^27^

### 2.10 Measurement of α-13′-COOH and Garcinoic Acid

Intracellular α-13′-COOH and GA in BMDMs were isolated as previously reported, with minor modifications. Briefly, cells were collected and resuspended in 0.25 M sodium acetate containing 10 mg/mL ascorbic acid, 10,000 U/mL beta-glucuronidase, and 200 U/mL sulfatase. After sonication for 2 min and incubation at 34 °C for 30 min, samples were acidified with concentrated acetic acid and ethanol, and a hexane/MTBE mixture (2:1, v/v) containing 50 mg/L BHT was added. Following vortexing for 1 min, samples were centrifuged at 3200 × *g* for 5 min at 10 °C and the organic phases were dried under nitrogen. MetOH-dH_2_O (3:1, v/v) containing 1 mg/L BHT was used to dissolve the samples prior to LC–MS/MS injection (AB Sciex 4000 QTRAP).^30^

The MS methods for the individual α-13′-COOH and GA standards were operated in negative ionization mode using dynamic multiple reaction monitoring (dMRM) coupled with an ESI source to determine the fragmentation patterns described in the literature.^7^ A Gemini C18 analytical column (10 cm × 2.0 mm, 3.0 μm particle size) was used, and samples were injected into the LC–MS/MS instrument (Agilent 6470 Triple Quadrupole). Mobile phase A was 100% dH_2_O, and mobile phase B was 100% MetOH containing 0.1% formic acid. The gradient began with 50% mobile phase B for 1 min and was thereafter ramped to 100% mobile phase B over an 8 min period and held for 7 min, returned to initial conditions in 1 min, and then re-equilibrated for 8 min. The column oven temperature was maintained at 40 °C, and the pump flow rate was maintained at 0.3 mL/min. The relative standard peak areas of α-13′-COOH and GA were determined and compared with the peak areas of samples.

### 2.11 Statistical Analysis

Prism 4 software (Graph-Pad, CA, USA) was used in performing statistical analysis. For the characterization results related to the synthesis of NPs, two-way ANOVA was used. Statistical significance in experiments using BMDMs was estimated using one-way ANOVA followed by Tukey’s multiple comparison test for multiple comparisons. The results were shown as mean ± standard deviation (SD), and a *p*-value less than 0.05 was considered statistically significant.

## 3 Results and Discussion

### 3.1 The S2P Peptide and DSPE-PEG-S2P Conjugate Were Successfully Synthesized and Characterized for Macrophage Targeting

α-13′-COOH is a long-chain metabolite of α-tocopherol produced during hepatic metabolism, whereas GA is a natural derivative of δ-tocotrienol isolated from *G. kola* nuts.^20,29^ Although studies have determined their protective effects in various disorders, both α-13′-COOH and GA are rapidly metabolized in the liver and detected at low concentrations, limiting their therapeutic potential.^6,7^ Therefore, strategies enhancing the half-life and directing to specific cells/tissue provide clinical potential. NPs are widely used in biomedical research as potential carriers, with their ability to protect bioactive molecules from degradation and prolong systemic circulation.^31^ NP-based carriers have been developed to target macrophages actively involved in the inflammatory response and deliver bioactive substances such as transcriptional inhibitors or anti-inflammatory agents to specific target sites/cells.^31^ In this study, macrophage-targeted NPs loaded with α-13′-COOH or GA were prepared to resolve inflammation and regulate immune responses that contribute to inflammatory diseases. Among emerging approaches, peptide-conjugated NPs represent an effective carrier system for targeted drug delivery.^32,33^ It has been determined that the stabilin-2 receptor is expressed in many macrophage populations,^34^ and the S2P peptide selectively binds to stabilin-2.^23,35^ In particular, Tao et al. showed that S2P-modified nanoparticles exhibit preferential uptake in macrophages and that this uptake is significantly reduced upon silencing of the stabilin-2 receptor, providing direct evidence of receptor-mediated internalization.^21^ Accordingly, the S2P peptide was utilized as a macrophage-targeting agent in this study. The peptide was purified (Figure S1), and its chemical characterization was performed using LC–MS/MS (Figure 2A). The molecular ion peak of the S2P peptide was observed at 1078.20 (*m*/*z*), closely matching its theoretical molecular weight of 1078.57 g/mol. Due to the presence of formic acid, +2 and +3 charged ion peaks were also detected at 539.60 and 360.10 (*m*/*z*), respectively, due to the presence of formic acid. After confirming successful synthesis, the S2P peptide was conjugated to maleimide-functionalized DSPE-PEG to form the DSPE-PEG-S2P-targeting conjugate, which was then chemically characterized using ^1^H NMR and FT-IR spectroscopies. In the ^1^H NMR spectrum (Figure 2B), the –NH amide protons of the S2P peptide appeared as a broad multiplet at 7.13–8.21 ppm. Also, the ester protons and –CH_2_ protons of the PEG polymer appeared at 4.04–4.19 ppm and 3.19–3.67 ppm, respectively. Moreover, the –CH_2_ and –CH_3_ protons of both DSPE lipid and S2P peptide were observed at 0.84–1.53 ppm. The FT-IR spectra of DSPE-PEG-Maleimide, S2P peptide, and DSPE-PEG-S2P conjugate were also analyzed to confirm the presence of characteristic functional groups from the precursor components in the conjugate structure (Figure 2C). In the FT-IR spectrum of the DSPE-PEG-S2P conjugate, O–H and N–H bonds, C–H and C–C bonds, and C–O bonds originating from both DSPE-PEG and S2P peptide were observed at 3273.65, 2881.07, and 1098.26 cm^–1^, respectively. Additionally, the C=O stretching peak of the maleimide functional group was detected in the FT-IR spectrum of DSPE-PEG-Maleimide at 1707.99 cm^–1^. However, after conjugation, this peak disappeared, confirming the successful formation of DSPE-PEG-S2P. Furthermore, C=O amide peaks from the S2P peptide in the conjugate were observed at 1632.08 cm^–1^. Overall, the presence of characteristic functional peaks from both DSPE-PEG and the S2P peptide validated the successful synthesis of DSPE-PEG-S2P as a targeting conjugate.

**Figure 2:**
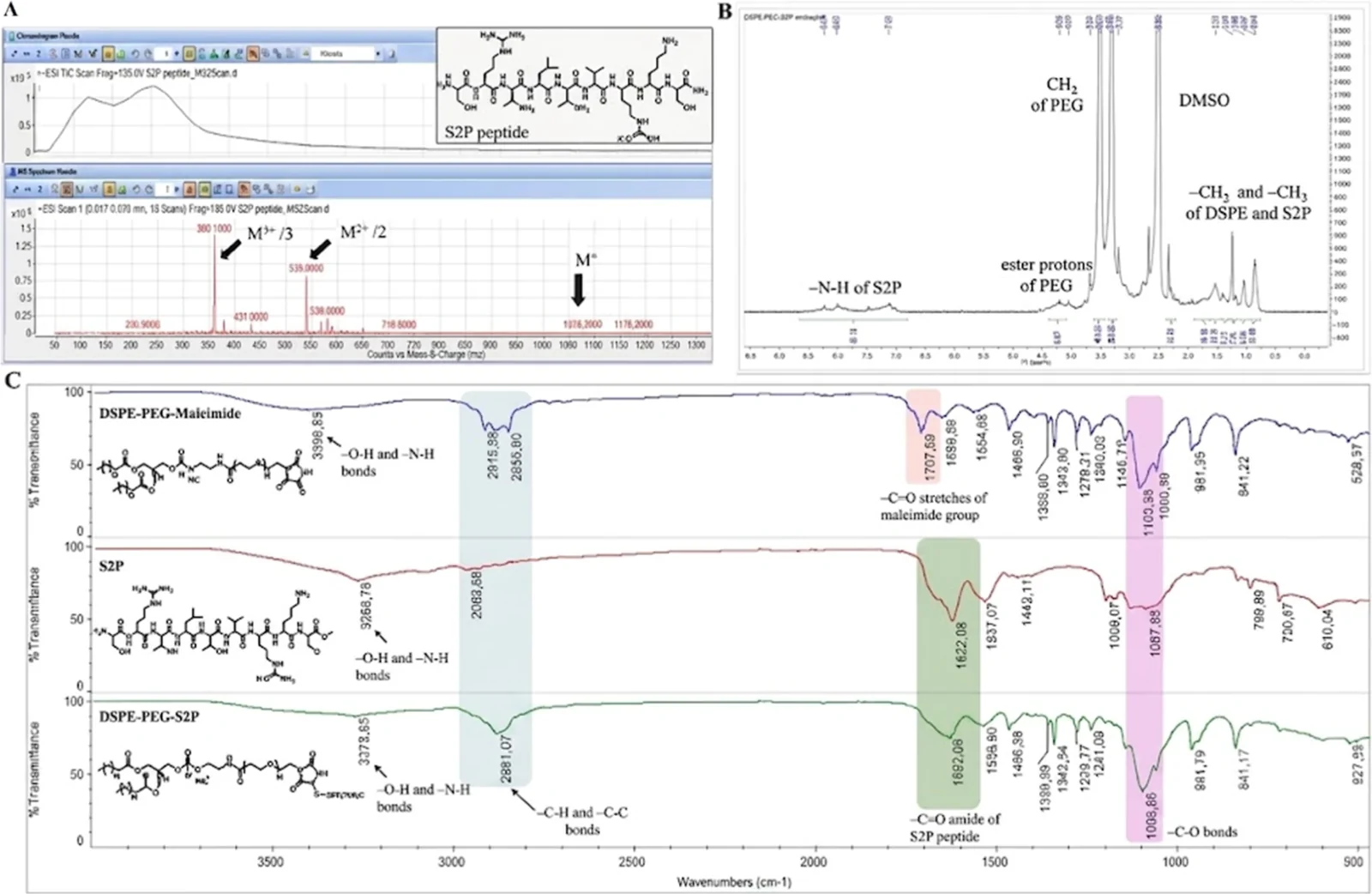
(A) LC–MS/MS results of the S2P peptide, (B) ^1^H NMR spectrum (in DMSO-*d*_6_) of the DSPE-PEG-S2P conjugate, and (C) FT-IR spectra of DSPE-PEG-Maleimide, the S2P peptide, and the DSPE-PEG-S2P conjugate.

### 3.2 Macrophage-Targeted Nanoparticles Containing α-13′-COOH or Garcinoic Acid Were Successfully Synthesized and Characterized

Macrophage-targeted NPs were synthesized following a reported method, with minor modifications based on optimization experiments.^23^ Previous studies have demonstrated that well-established PEGylated PLGA systems can maintain stable size distribution and dispersion behavior in serum-containing media, supporting their suitability for biological applications and having a higher statistical probability of multiple passes through the target tissue due to reduced nonspecific interactions, increasing the likelihood of successful endothelial interaction despite high-pressure flow.^36^ In brief, the organic phase, containing hydrophobic PLGA and drug molecules, was added dropwise to the aqueous phase. Due to the hydrophobicity-based physical interactions induced, self-assembling PLGA NPs with hydrophobic drug molecules encapsulated in their core were formed. The synthesized macrophage-targeted NPs were then characterized in terms of their hydrodynamic diameter, surface charge, and DEE in comparison with the nontargeted ones (Table 1). The hydrodynamic diameter of noncoated NPs (NP1) was measured as 165.0 ± 4.2 nm. After coating with the PEGylated lipid, the NP size increased, ranging from 227.9 ± 8.6 nm to 335.4 ± 21.4 nm after S2P conjugation. The increase in NP size is likely due to the addition of hydrophilic PEG chains on the surface (Figures S3 and S4). However, all NPs exhibited a monodisperse distribution with PDI values below 0.213 ± 0.110, the size within the desired range for blood circulation.^37,38^ Furthermore, nontargeted NP1, NP2, NP4, and NP6 exhibited negative surface charge values ranging from −26.90 ± 0.85 to −22.70 ± 4.03 mV. Following the conjugation of the S2P peptide as a macrophage-targeting agent, the surface charge of the NPs shifted toward less negative values, ranging from −20.70 ± 0.50 to −13.60 ± 0.72 mV. This change in surface charges could be attributed to the successful attachment of the positively charged peptide onto the NP surface (Figures S5 and S6). The presence of the peptide was further confirmed by measuring its concentration on the NPs using a Nanodrop device. The S2P concentrations for NP3 and NP5 were found to be 17.54 ± 2.45 and 17.34 ± 5.26 μg/mL, respectively. This result demonstrates that there was almost no loss of the targeting peptide during the drug loading process. The DEE of macrophage-targeted NPs was comparable to that of nontargeted NPs, with values ranging from 80.00% ± 0.55 to 80.40% ± 0.23 for α-13′-COOH and 18.82% ± 0.69 to 20.52% ± 0.74 for GA. This indicates that the addition of the targeting peptide did not significantly affect the drug encapsulation process. Notably, DEE was higher for α-13′-COOH, likely due to differences in chemical structure and hydrophobicity between the two drugs.

**Table 1:** Formulation Details and Characterization Results of NPs, Including Their Peptide Concentration, Particle Size, PDI Value, Zeta Potential, and DEE

PLGA NPs	DSPE-PEG coating	α-13′-COOH	GA^a^	S2P	S2P concentration (μg/mL)^b^	size^b^ ^,^ ^c^ (nm)	PDI^b^ ^,^ ^d^	zeta potential^b^ (mV)	DEE^b^ ^,^ ^e^ (%)
NP1	-	-	-	-	-	165.0 ± 4.2	0.094 ± 0.091	–24.20 ± 4.00	-
NP2	+	-	-	-	-	234.8 ± 7.4	0.213 ± 0.110	–25.10 ± 3.13	-
NP3	+	-	-	+	17.54 ± 2.45	321.1 ± 12.8	0.115 ± 0.073	–16.70 ± 4.88	-
NP4	+	+	-	-	-	227.9 ± 8.6	0.089 ± 0.061	–22.70 ± 4.03	80.40 ± 0.23
NP5	+	+	-	+	17.34 ± 5.26	335.4 ± 21.4	0.118 ± 0.172	–13.60 ± 0.72	80.00 ± 0.55
NP6	+	-	+	-	-	274.0 ± 10.2	0.171 ± 0.094	–26.90 ± 0.85	20.59 ± 0.74
NP7	+	-	+	+	^f^	253.2 ± 8.8	0.136 ± 0.045	–20.70 ± 0.50	18.82 ±0.69

a Garcinoic acid.

b Data are expressed as mean ± SD (*n* = 3).

c Size values are given in diameter.

d Polydispersity index.

e Drug-encapsulation efficiency.

f It could not be determined due to interference in the measurements obtained from the Nanodrop, which operates based on absorbance.

The diameter and morphology of the NPs were further analyzed using both SEM and TEM (Figure 3). Nanosized spherical NPs with smooth, distinct morphologies were observed, along with a coating layer surrounding them. However, the hydrodynamic diameter of NPs that were measured with TEM was smaller than that of NPs that were measured with DLS because of dehydration.^39^ NPs were synthesized and stored in water. However, to perform the SEM and TEM analyses, the NP solutions were dropped on grids, and they were allowed to dry for 2 days under sterile conditions. After the water evaporated, the analyses were conducted with a slight reduction in the observed size values.

**Figure 3:**
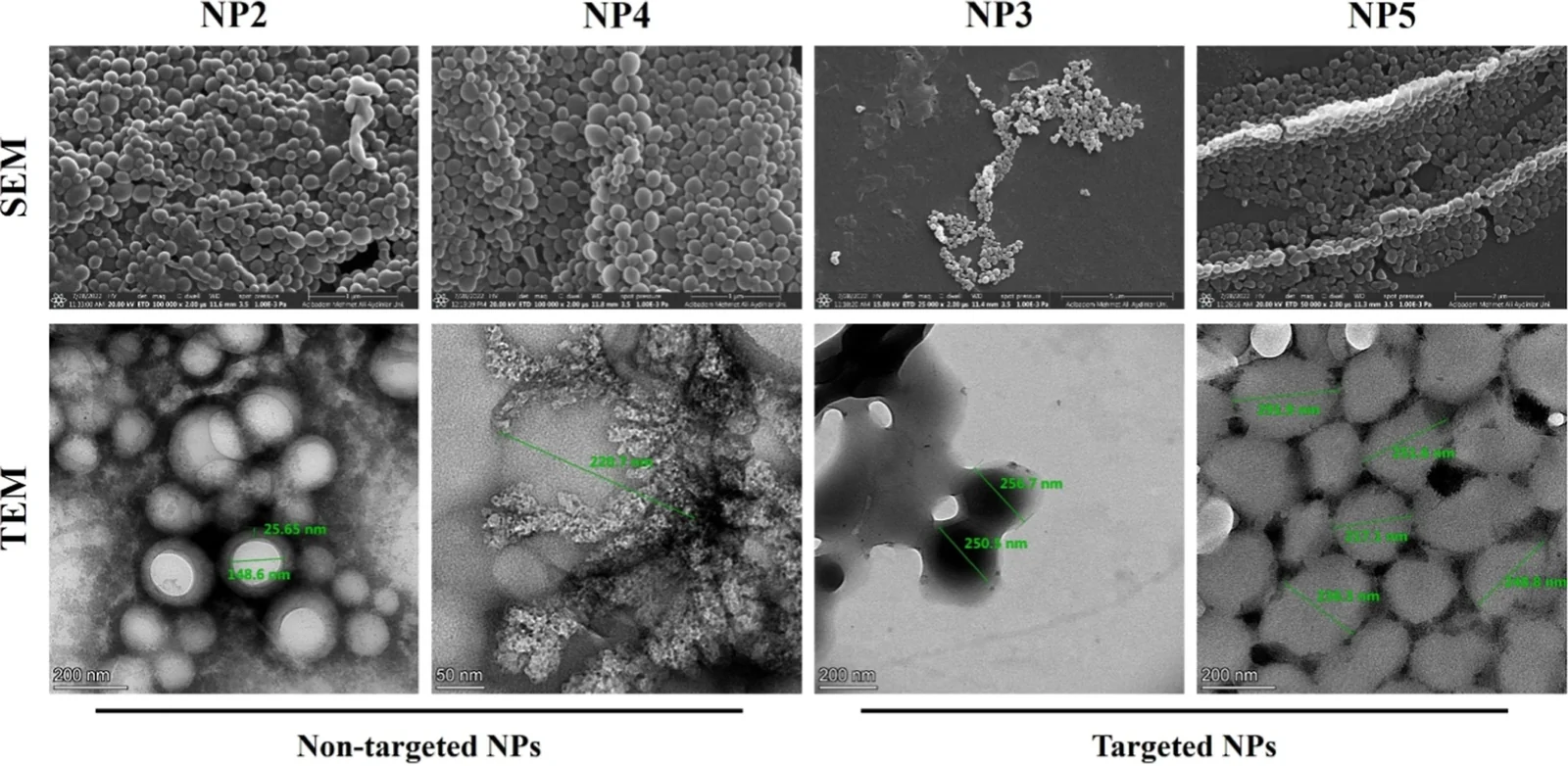
SEM and TEM images of nontargeted (NP2 and NP4) and targeted (NP3 and NP5) NPs (NP2: PLGA NPs; NP3: S2P-PLGA NPs; NP4: α-13′-COOH-PLGA NPs; NP5: S2P-α-13′-COOH-PLGA NPs).

Furthermore, the stability of NP2, NP4, and NP6 was evaluated by incubating them in pH 7.4 PBS at RT and 37 °C. As described in the methodology, our nanoparticles were formulated with a DSPE-PEG coating, where PEGylation provides a hydrophilic steric barrier at the nanoparticle surface, which is known to limit protein adsorption and reduce aggregation in serum-containing environments.^40,41^ Figure 4 demonstrates that the hydrodynamic diameters of all NPs remained statistically stable during the first 2 days at both temperatures, regardless of the encapsulated drug molecule. Notably, NP6, loaded with GA as the drug molecule, exhibited greater stability compared to α-13′-COOH-loaded NPs. This enhanced stability can be attributed to the lower DEE of GA-loaded NPs than α-13′-COOH-loaded ones. The lower encapsulation reduced the rate of sudden drug release, thereby increasing stability. After day 2, a gradual increase in NP size was observed, which could result from swelling in the PBS solution, maybe leading to the partial separation of polymer chains.^39^

**Figure 4:**
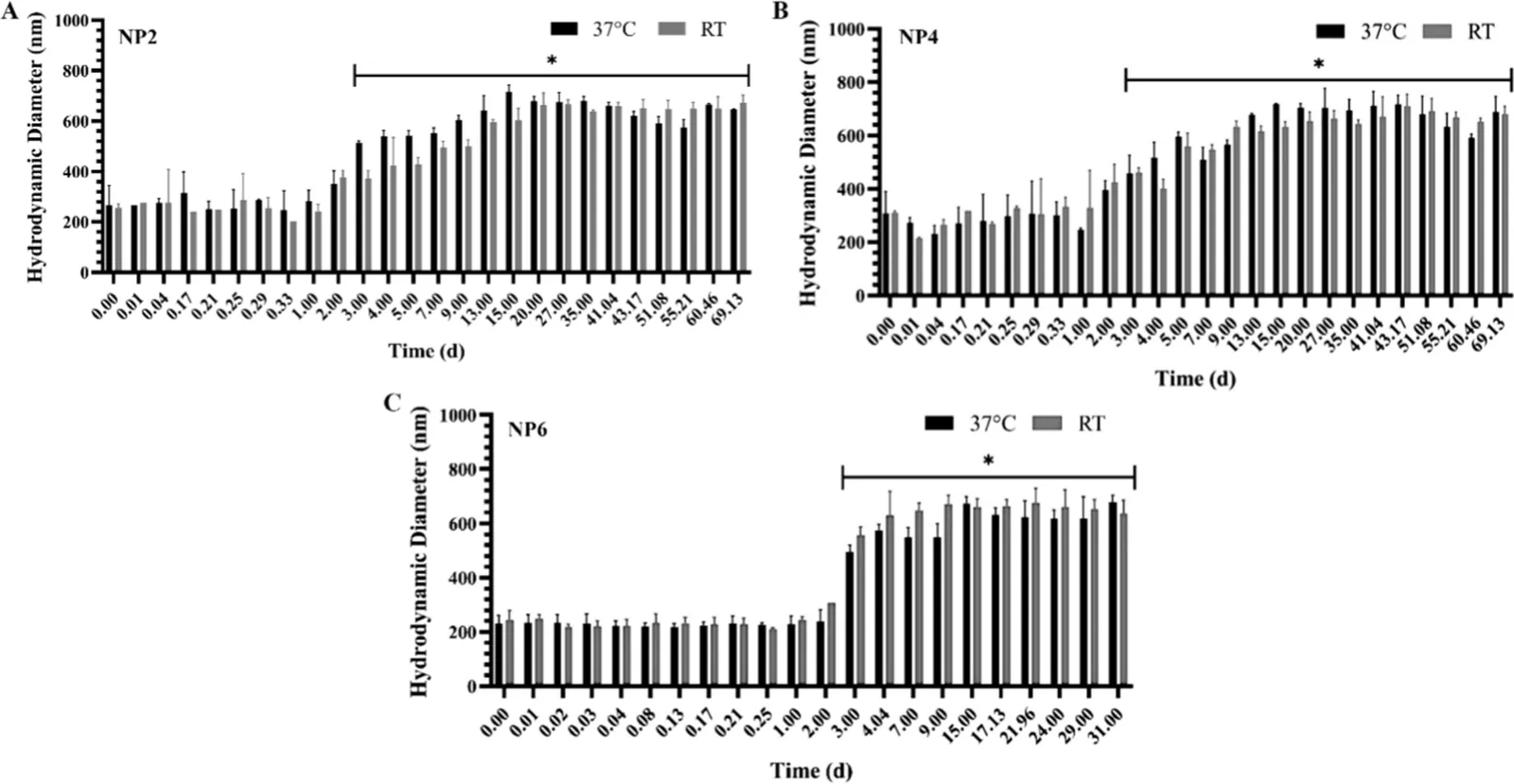
Stability analysis of NP2 (A), NP4 (B), and NP6 (C). *p < 0.0001 for the hydrodynamic diameter of NPs with respect to their diameter during the first 2 days. Stability results are expressed as mean ± SD (*n* = 3) (NP2: PLGA NPs; NP4: α-13′-COOH-PLGA NPs; NP6: GA-PLGA NPs).

Finally, the release rates of α-13′-COOH from NP4 and GA from NP6 were evaluated in two different environments: pH 7.4 PBS solution to simulate healthy body conditions and pH 5.5 acetate buffer solution to mimic slightly acidic inflammatory conditions. The data obtained from LC–MS/MS analysis were then fitted to the Korsmeyer–Peppas model, revealing sustained and prolonged drug release from NPs (Figure 5). Consistent with previous reports, the ester bonds in the PLGA polymer degrade more rapidly under acidic conditions.^42^ Therefore, for both drug molecules, drug release from NPs was found to be significantly higher at pH 5.5 compared to a neutral environment. After 21 days, α-13′-COOH release from NP4 reached approximately 86% at pH 5.5, while it was only around 25% at pH 7.4. Similarly, GA release from NP6 reached nearly 72% at pH 5.5 after 21 days, while it was only about 4% at pH 7.4 (Figures S7 and S8). This result clearly demonstrates enhanced drug release in inflammatory conditions compared to healthy body fluids.^43^ Moreover, the drug release rate from NP6 was slower than that from NP4. This difference could be attributed to the lower DEE of GA-loaded NPs compared to α-13′-COOH-loaded NPs. Overall, prolonged and pH-responsive drug release was successfully achieved for both α-13′-COOH and GA-loaded NPs.

**Figure 5:**
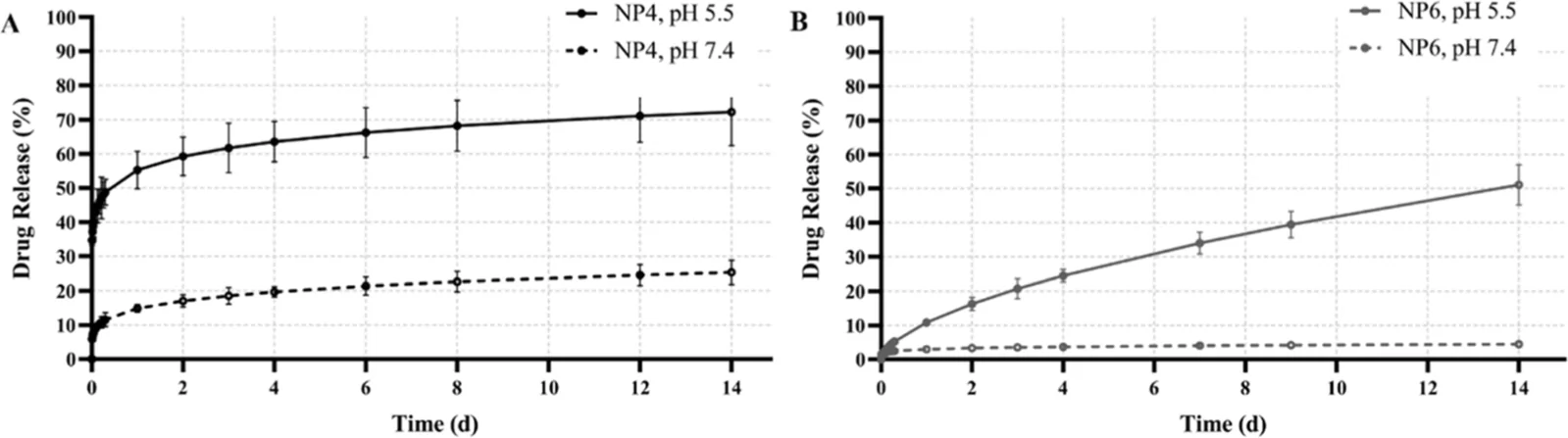
Drug release profiles of NP4 (A) and NP6 (B) over 14 days, fitted using the Korsmeyer–Peppas model. Drug release results are reported as mean ± SD (*n* = 3).

Additionally, the fitting parameters (*K*, *n*, *R* ^2^) and the corresponding diffusion types are summarized in Table 2. According to the literature, a higher K value indicates faster drug release.^44^ As shown in Table 2, both NP4 and NP6 exhibited higher K values at pH 5.5 compared to pH 7.4, indicating enhanced drug release from the NPs in acidic inflammatory environments relative to normal physiological conditions. Furthermore, the diffusion type could be determined from the *n*-value obtained from the Korsmeyer–Peppas model. The *n*-value was obtained as smaller than 0.203 for NP4 at both pH values and for NP6 at pH 7.4. However, the n value was found to be 0.587 for NP6 at pH 5.5. In the literature, an *n* value ≤0.450 indicates that drug release from polymeric NPs follows Fickian diffusion, while an *n* value between 0.450 and 0.890 suggests nonanomalous (non-Fickian) diffusion.^45^ Lastly, the correlation coefficients (*R* ^2^) for the Korsmeyer–Peppas model were found to be greater than 0.900, showing the release kinetics align well with this model. All the obtained data indicates that DSPE-PEG-coated, macrophage-targeted, α-13′-COOH or GA-containing biocompatible and biodegradable PLGA NPs were successfully synthesized in a monodisperse manner, and their characterization tests were completed prior to in vitro cell culture studies.

**Table 2:** Mathematical Parameters of the Drug Release Data Fitted to the Korsmeyer–Peppas Model

PLGA NPs	pH	*K* ^a^	*n* ^b^	*R* ^2^ ^c^	diffusion type
NP4	5.5	0.552	0.102	0.972	Fickian
NP4	7.4	0.148	0.203	0.975	Fickian
NP6	5.5	0.108	0.587	0.992	non-Fickian
NP6	7.4	0.030	0.153	0.900	Fickian

a Korsmeyer–Peppas model release rate constant.

b Diffusion index.

c Fitting the correlation coefficient.

### 3.3 Macrophage Targeting of Nanoparticles Enhances the Cellular Levels of α-13′-COOH and Garcinoic Acid without Affecting Cell Viability and Morphology

Following NP synthesis and characterization, in vitro cell culture experiments were conducted to evaluate macrophage viability and intracellular accumulation of α-13′-COOH or GA-loaded nanoparticles (Figure 6). Due to phenotypic differences and in vivo heterogeneity, studies indicate that the findings obtained with transformed monocyte/macrophage cell lines may differ from those observed in primary cell behavior.^46,47^ In the present study, we used BMDMs (bone marrow-derived macrophages) as a primary macrophage cell line. BMDMs were treated for 24 h with various concentrations of α-13′-COOH and GA-loaded NPs, and no significant cytotoxic effect was observed (Figure 6A). The effect of NPs at various concentrations of α-13′-COOH and GA was also investigated in the context of α-13′-COOH and GA accumulation. As shown in Figure 6B, NP administration significantly increased α-13′-COOH and GA levels, while a marked dose-dependent enhancement was observed in macrophage-targeted ones. Therefore, the optimum concentration for the following experiments was determined as 0.5 μM, and NP accumulation was further confirmed using electron microscopy. Consistent with previous observations, NP administration had no effect on macrophage ultrastructural morphology. Nontargeted formulations remained predominantly in the extracellular space, whereas macrophage-targeted nanoparticles loaded with either α-13′-COOH or GA exhibited clear intracellular internalization within the cytoplasm (Figure 6C).^48^ Consequently, the higher NP accumulation and a dose-dependent increase of α-13′-COOH and GA levels were observed in macrophage-targeted NP-administered groups, compared to the nontargeted ones, which points out to the uptake of S2P-functionalized PLGA–PEG nanoparticles via a ligand–receptor-based internalization.

**Figure 6:**
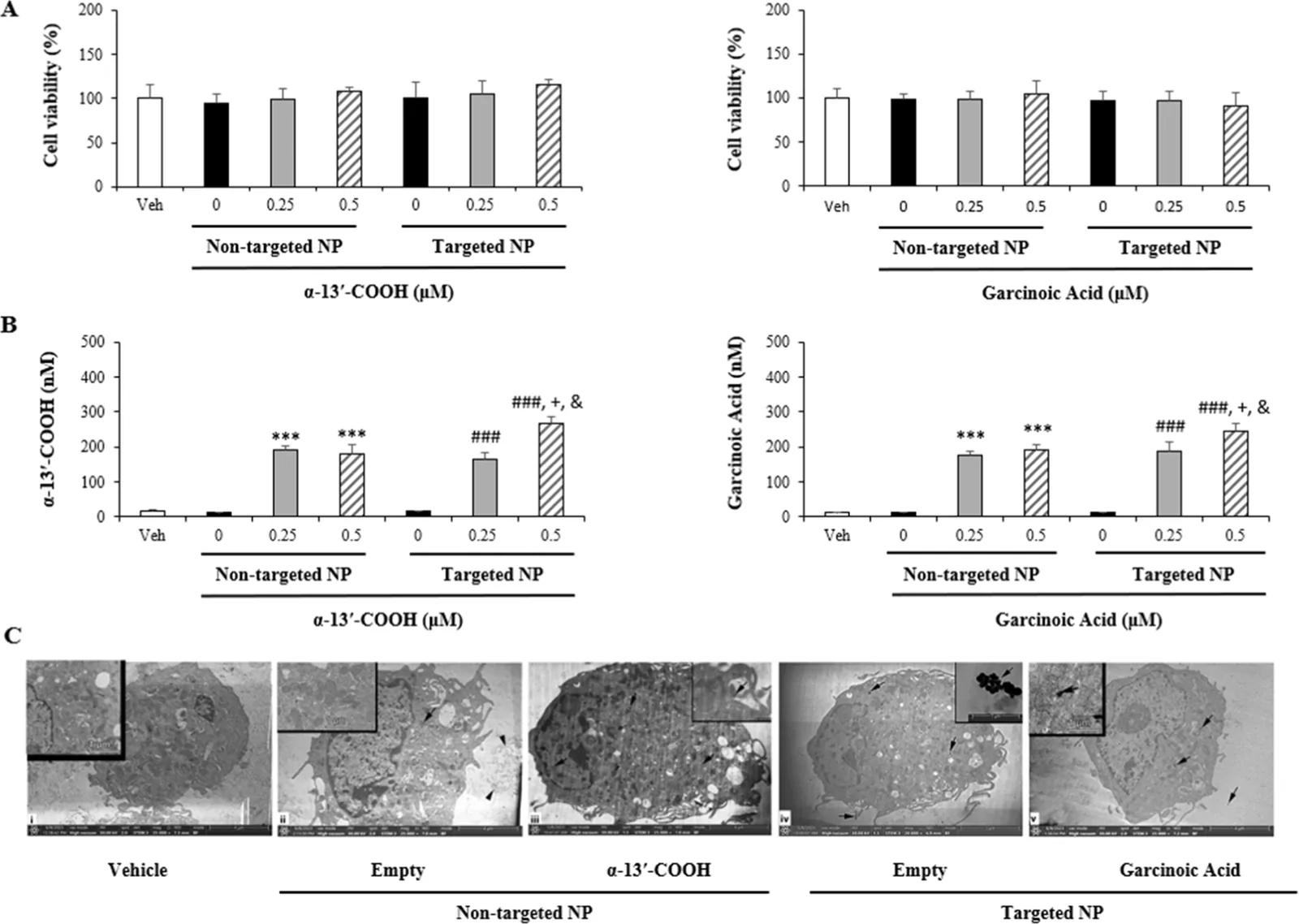
(A,B) BMDMs were treated with different concentrations of α-13′-COOH or GA-loaded NPs (0–5 μM) for 24 h. (A) Viability was analyzed by an MTT assay. (B) The levels of α-13′-COOH and GA were measured by LC–MS/MS. (C_i–v_) BMDMs were treated with α-13′-COOH or GA-loaded NPs at 0.5 μM for 24 h. Representative electron microscopic images show regular ultrastructural morphology in all groups. (C_ii,iii_) In the vehicle group, normal ultrastructural morphology was observed. Cytoplasmic organization with intact mitochondria (inset) appeared normal. In the nontargeted empty nanoparticle group, particles were mainly located outside the cells (arrowhead), with some observed in the cytoplasm (arrow); overall morphology remained normal (inset). In the nontargeted α-13′-COOH-loaded group, nanoparticle accumulations were detected in the cytoplasm (arrow), and some particles were localized within lysosomes (inset, arrow). In the targeted empty nanoparticle group, particles were observed in the cytoplasm, while others were located outside the cells (inset, arrow). In the GA-loaded targeted nanoparticle group, nanoparticles were present in the cytoplasm, with some accumulations noted in close proximity to mitochondria (inset). Few nanoparticles in the cytoplasm (arrow) and numerous nanoparticles outside (arrowhead) are seen in BMDMs incubated with nontargeted NPs. (C_iv,iv_) Data are expressed as mean ± SD. One-way ANOVA, Tukey’s multiple comparison test. ****p < 0.001 vs nontargeted empty NP group, ^###^ *p* < 0.001 vs targeted empty NP group, ^+^ *p* < 0.05 vs targeted 0.25 μM NP group, ^&^ *p* < 0.05 vs nontargeted 0.5 μM NP group (*n* = 3).

## 4 Conclusion

As the body’s first line of defense, macrophages are essential not only for immunity but also for development, homeostasis, and tissue repair. Considering the importance of α-13′-COOH and GA in modulating signaling pathways, strategies directing immune cells by NP-mediated drug delivery hold promise as a crucial therapeutic goal. In this context, we successfully developed α-13′-COOH- or GA-loaded, macrophage-targeted NPs, which were characterized and optimized based on their hydrodynamic diameter, surface charge, drug encapsulation efficiency, morphology, stability, and drug release profile. Current findings of in vitro studies demonstrated that macrophage-targeted NPs accumulated to a greater extent within the cells than nontargeted ones, and cellular α-13′-COOH/GA levels increased for targeted NPs in a dose-dependent manner without affecting cell viability. Observation of NP accumulation and α-13′-COOH or GA increase, mostly in macrophage-targeted ones, is significant in revealing the success of our macrophage targeting strategy. The demonstrated potential of PEGylated PLGA nanoparticles for delivering these vitamin E derivatives to macrophages in a dose-dependent manner obviously contributes to the development of innovative strategies to treat macrophage-associated inflammatory diseases. Further preclinical studies are required to evaluate the selectivity and therapeutic efficacy of this delivery system.

## Supplementary Material



## Notes

Figure S1: Prep-HPLC analysis of the S2P peptide; Figure S2: FT-IR spectra of α-13′-COOH, PLGA, NP1, NP2, and NP4 (NP1: noncoated NP, NP2: PLGA NP, NP4: α-13′-COOH PLGA NP); Figure S3: hydrodynamic diameter and PDI measurements of nontargeted NP1 (noncoated NPs), NP2 (PLGA NP), NP4 (α-13′-COOH PLGA NP), and NP6 (GA-PLGA NP) via DLS; Figure S4: hydrodynamic diameter and PDI measurements of targeted NP3 (S2P-PLGA NP), NP5 (S2P-α-13′-COOH PLGA NP), and NP7 (S2P-GA-PLGA NP) via DLS; Figure S5: zeta potential measurements of nontargeted NP1 (noncoated NPs), NP2 (PLGA NP), NP4 (α-13′-COOH PLGA NP), and NP6 (GA-PLGA NP); Figure S6: zeta potential measurements of targeted NP3 (S2P-PLGA NP), NP5 (S2P-α-13′-COOH PLGA NP), and NP7 (S2P-GA-PLGA NP); Figure S7: primary drug release profiles of NP4 and NP6 studied over 22 days; Figure S8: drug release profiles of NP4 and NP6 over 22 days, fitted using the Korsmeyer–Peppas model; and Figure S9: standard calibration curve used for S2P peptide quantification (PDF)

## Abbreviations

α-13′-COOH: alpha-13′-carboxychromanol
BMDM: bone marrow-derived macrophages
δ-T3: delta-tocotrienol
DLS: dynamic light scattering
GA: garcinoic acid
NP: nanoparticle
PEG: polyethylene glycol
PLGA: poly­(lactic-*co*-glycolic acid)
SEM: scanning electron microscopy
TEM: transmission electron microscopy
