# Supplementary material for: Development and In Vitro Efficacy of Macrophage-Targeted Nanoparticles Encapsulating Vitamin E Derivatives Garcinoic Acid and α‑13′-Carboxychromanol
Source: ACS Omega. 2026 Aug 21;11(35):52488–98. doi: 10.1021/acsomega.6c01032 (PMC13563993; doi:10.1021/acsomega.6c01032)
Supplement: Supplementary file 1 [file ao6c01032_si_001.pdf]

Development and *in vitro* Efficacy of Macrophage-Targeted Nanoparticles Encapsulating Vitamin E Derivatives Garcinoic Acid and α-13′-Carboxychromanol

Deniz Gol[1#], Eda Ilayda Talaz[2,3#], Seher Mese-Tayfur[2,3], Merve Acikel Elmas[4], Antimo Gioiello[5], Francesco Galli[6], Nesrin Kartal Ozer[7,8], Erdi Sozen[2,9*], Ozgul Gok[1,10,11*]

[1]Department of Biomedical Engineering, Graduate School of Natural and Applied Sciences, Acibadem Mehmet Ali Aydinlar University, 34752, Istanbul, Türkiye

[2]Department of Biochemistry, Faculty of Medicine, Marmara University, 34854, Istanbul, Türkiye

[3]Institute of Health Sciences, Marmara University, 34854, Istanbul, Türkiye

[4]Department of Histology and Embriology, School of Medicine, Acıbadem Mehmet Ali Aydınlar University, 34752, Istanbul, Türkiye

[5]Laboratory of Medicinal and Advanced Synthetic Chemistry (Lab MASC), Department of Pharmaceutical Sciences, University of Perugia, 06122, Perugia, Italy

[6]Human Nutrition and Nutrigenomics Lab, Dept of Pharmaceutical Sciences, University of Perugia, 06122, Perugia, Italy

[7]Department of Biochemistry, Faculty of Medicine, Uskudar University, 34662, Istanbul, Türkiye

[8]Metabolic and Inflammatory Diseases Research Center (METIFLAM), Uskudar University, 34662, Istanbul, Türkiye

[9]Genetic and Metabolic Diseases Research and Investigation Center (GEMHAM), Marmara University, 34854, Istanbul, Türkiye

[10]Department of Biomedical Engineering, Faculty of Engineering and Natural Sciences, Acibadem Mehmet Ali Aydinlar University, 34752, Istanbul, Türkiye

[11]Bogazici University, Center for Targeted Therapy Technologies, Istanbul 34684, Türkiye

[#]These authors contributed equally

*Corresponding author: Ozgul Gok, Department of Biomedical Engineering, Faculty of Engineering and Natural Sciences, Acibadem Mehmet Ali Aydinlar University, Istanbul 34752, Türkiye. ozgul.gok@acibadem.edu.tr

*Co-corresponding author: Erdi Sozen, Department of Biochemistry, Faculty of Medicine, Marmara University, Maltepe, Istanbul, 34854, Turkey. Tel/Fax: + 90 216 7775623, erdi.sozen@marmara.edu.tr

Table of Contents

Figure S1. Prep-HPLC analysis of the S2P peptide. 3

Figure S2. FT-IR spectra of **(A)** α-13′-COOH, **(B)** PLGA, **(C)** NP1, **(D)** NP2, and **(E)** NP4. (**NP1**: non-coated NP, **NP2**: PLGA NP, **NP4**: α-13′-COOH PLGA NP)..................................4

Figure S3. Hydrodynamic diameter and PDI measurements of non-targeted NP1 (non-coated NPs), NP2 (PLGA NP), NP4 (α-13′-COOH PLGA NP), and NP6 (GA-PLGA NP) via DLS. .5

Figure S4. Hydrodynamic diameter and PDI measurements of targeted NP3 (S2P-PLGA NP), NP5 (S2P-α-13′-COOH PLGA NP), and NP7 (S2P-GA-PLGA NP) via DLS. 5

Figure S5. Zeta potential measurements of non-targeted NP1 (non-coated NPs), NP2 (PLGA NP), NP4 (α-13′-COOH PLGA NP), and NP6 (GA-PLGA NP)...6

Figure S6. Zeta potential measurements of targeted NP3 (S2P-PLGA NP), NP5 (S2P-α-13′-COOH PLGA NP), and NP7 (S2P-GA-PLGA NP). 6

Figure S7. Primary drug release profiles of NP4 and NP6 studied over 22 days. Data are presented as mean ± SD (n = 3). 7

Figure S8. Drug release profiles of NP4 and NP6 over 22 days, fitted using the Korsmeyer-Peppas model. Data are presented as mean ± SD (n = 3). 7

Figure S9. Standard calibration curve used for S2P peptide quantification.. 8

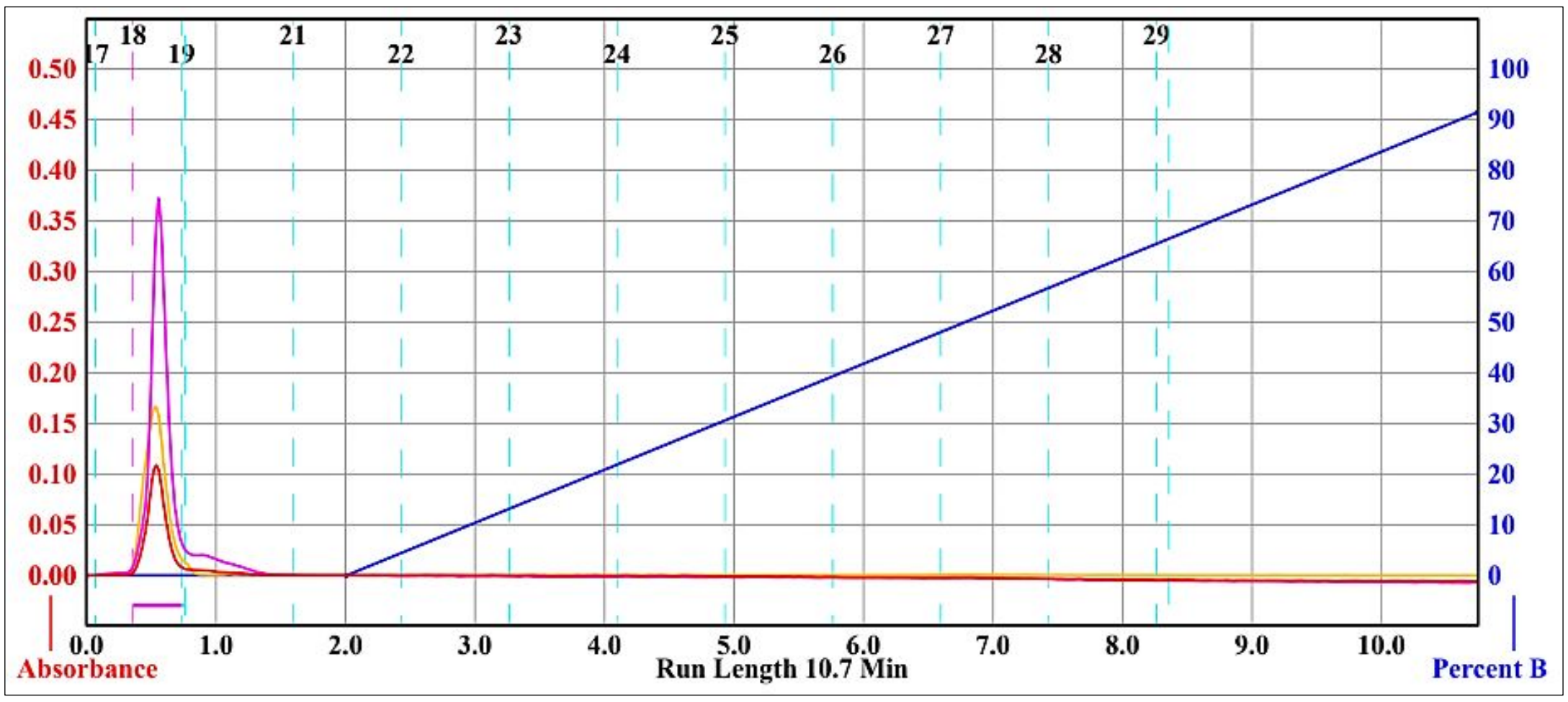

Fig. S1 Prep-HPLC analysis of the S2P peptide.

The synthesized S2P peptide was first run under 100% ddH_2O for 2 min, followed by a linear gradient increase from 0 to 100% acetonitrile over 10 min. The relevant pure peptide peaks, identified as the 17th, 18th, and 19th peaks, were collected between 0-1 min in 100% ddH_2O.

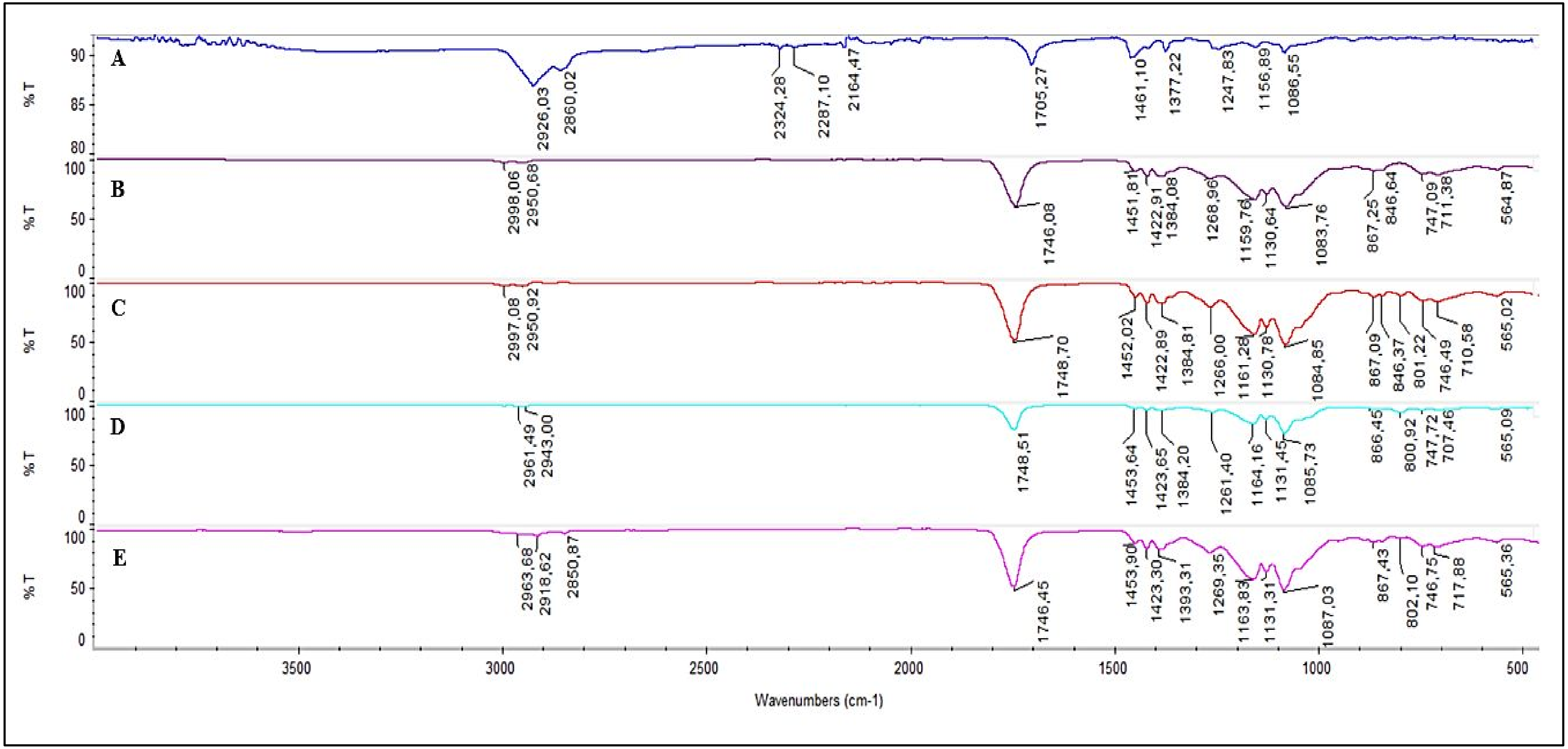

Fig. S2 FT-IR spectra of **(A)** α-13′-COOH, **(B)** PLGA, **(C)** NP1, **(D)** NP2, and **(E)** NP4. (**NP1**: non-coated NP, **NP2**: PLGA NP, **NP4**: α-13′-COOH PLGA NP)

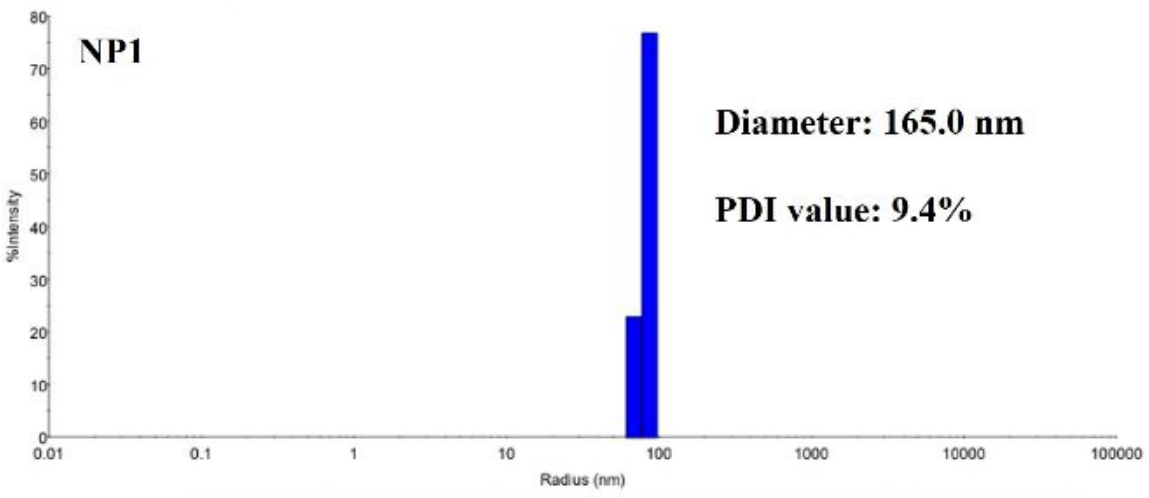

	Radius (nm)	Mw-R (kDa)	%Pd	%Intensity	%Mass	%Number
Peak 1	82.5	102702.0	9.4	100.0	100.0	100.0

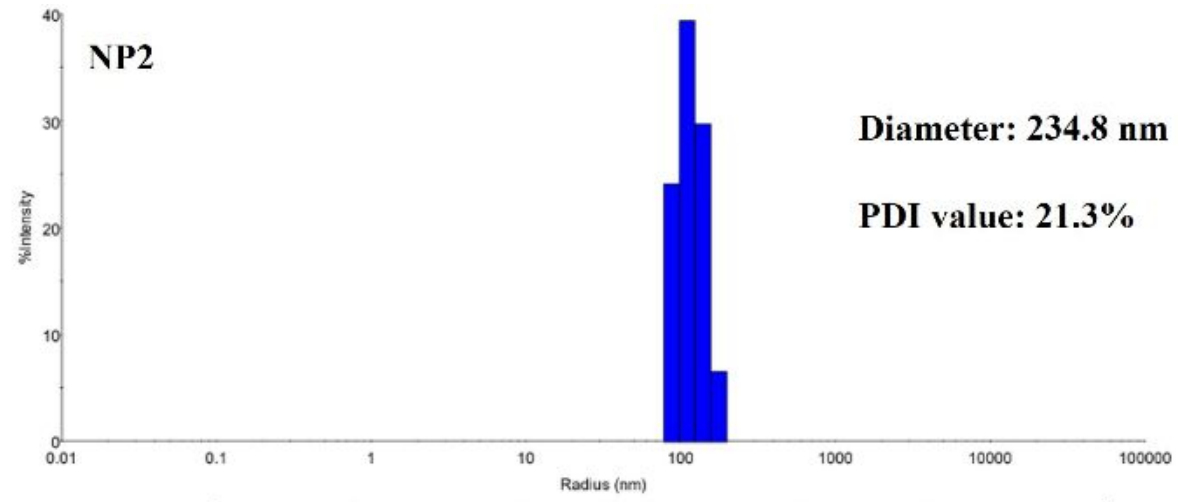

	Radius (nm)	Mw-R (kDa)	%Pd	%Intensity	%Mass	%Number
Peak 1	117.4	234356.6	21.3	100.0	100.0	100.0

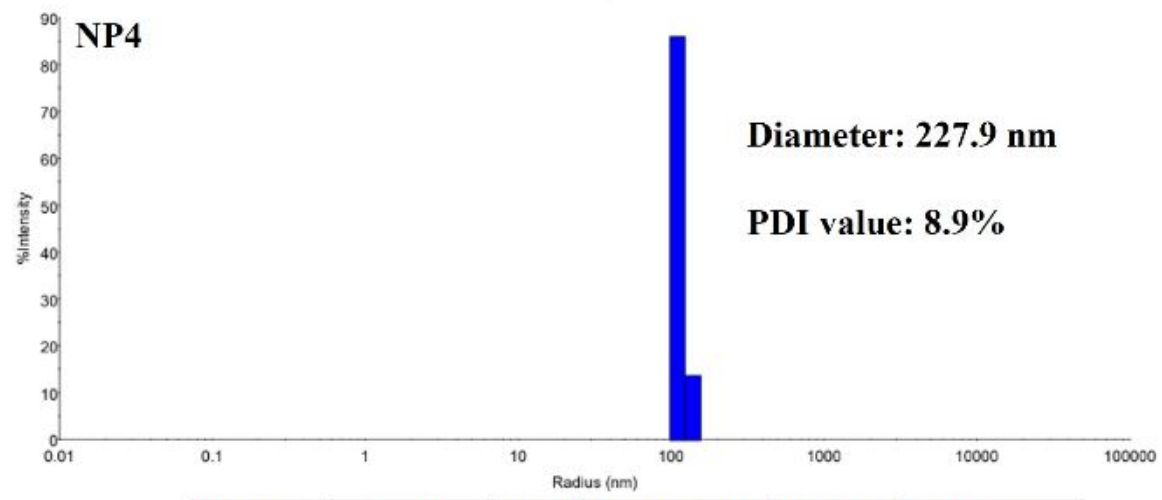

	Radius (nm)	Mw-R (kDa)	%Pd	%Intensity	%Mass	%Number
Peak 1	113.9	218476.9	8.9	100.0	100.0	100.0

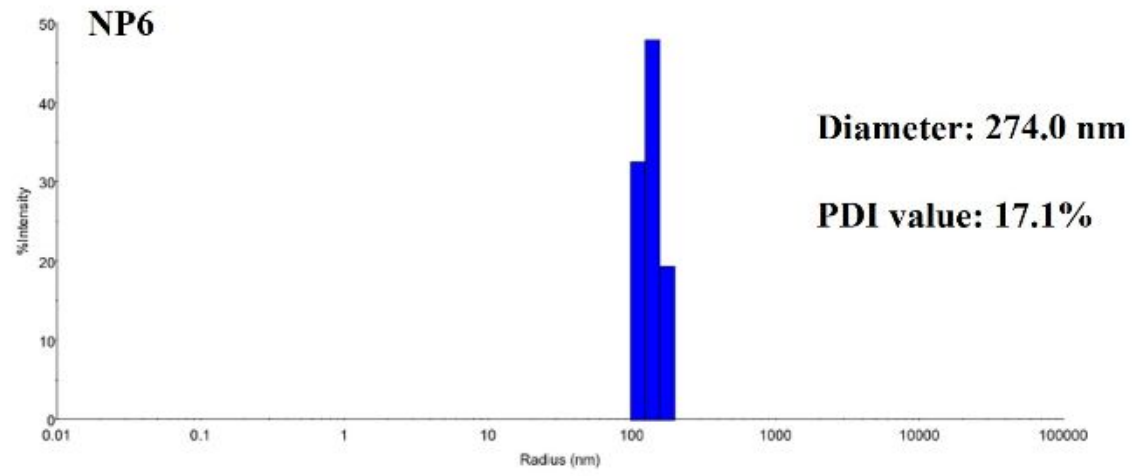

	Radius (nm)	Mw-R (kDa)	%Pd	%Intensity	%Mass	%Number
Peak 1	137.0	336132.0	17.1	100.0	100.0	100.0

Fig. S3 Hydrodynamic diameter and PDI measurements of non-targeted NP1 (non-coated NP), NP2 (PLGA NP), NP4 (α-13′-COOH PLGA NP), and NP6 (GA-PLGA NP) via DLS.

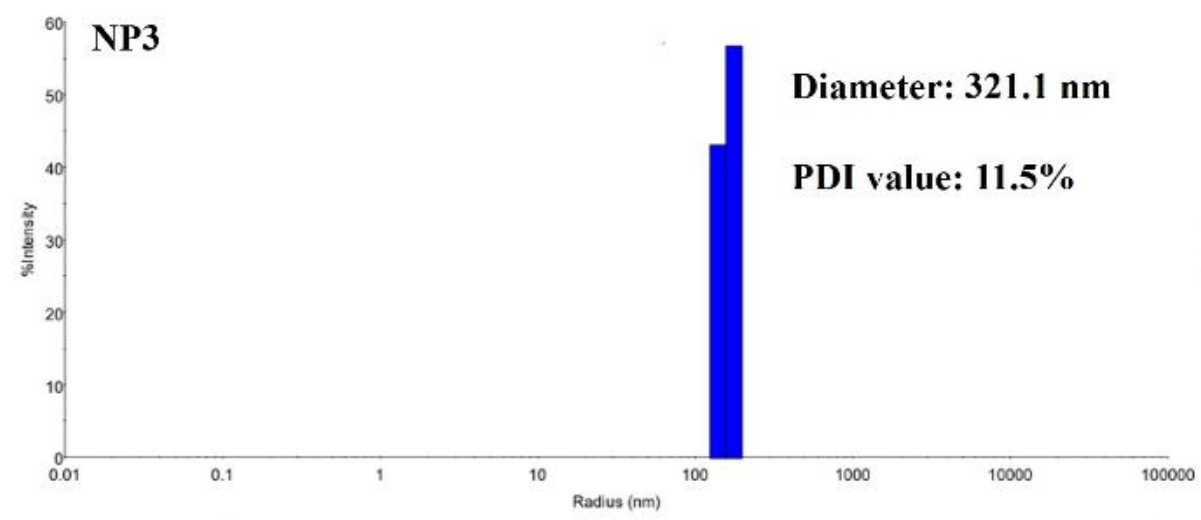

	Radius (nm)	Mw-R (kDa)	%Pd	%Intensity	%Mass	%Number
Peak 1	160.5	487351.5	11.5	100.0	100.0	100.0

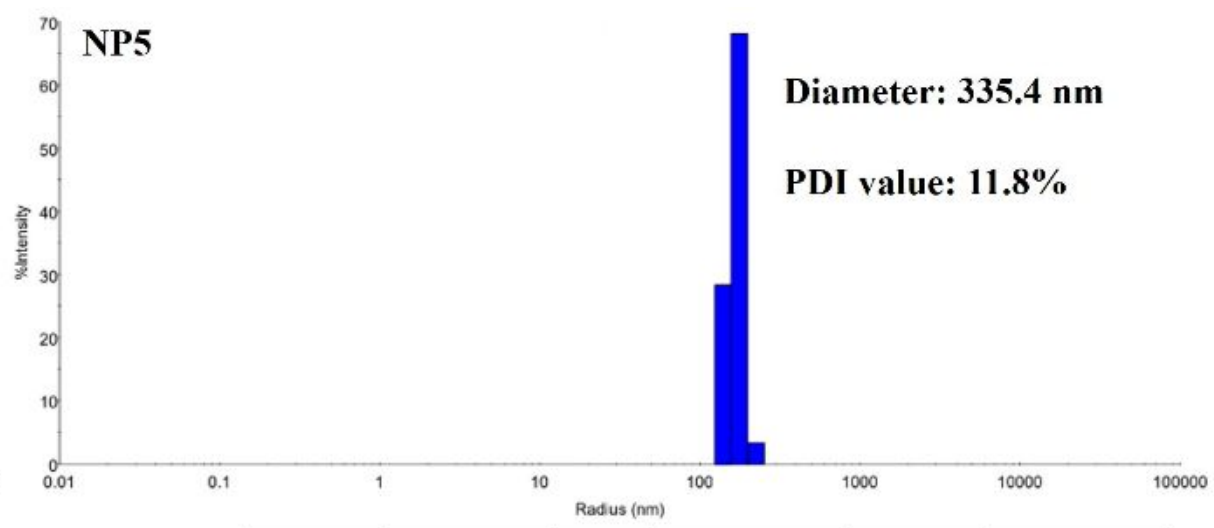

	Radius (nm)	Mw-R (kDa)	%Pd	%Intensity	%Mass	%Number
Peak 1	167.7	539507.7	11.8	100.0	100.0	100.0

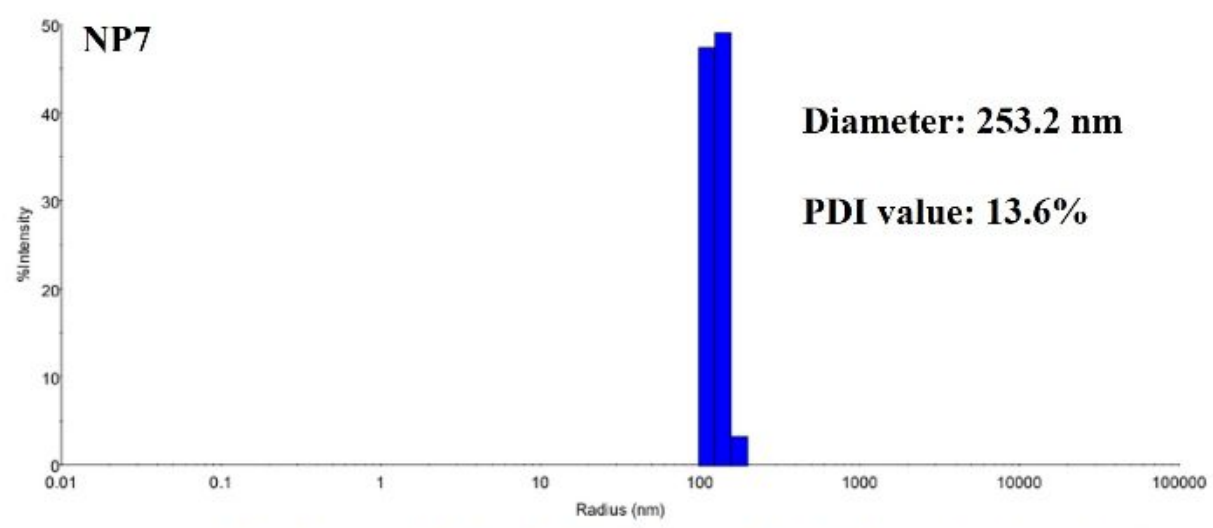

	Radius (nm)	Mw-R (kDa)	%Pd	%Intensity	%Mass	%Number
Peak 1	126.6	279436.3	13.6	100.0	100.0	100.0

Fig. S4 Hydrodynamic diameter and PDI measurements of targeted NP3 (S2P-PLGA NP), NP5 (S2P-α-13′-COOH PLGA NP), and NP7 (S2P-GA-PLGA NP) via DLS.

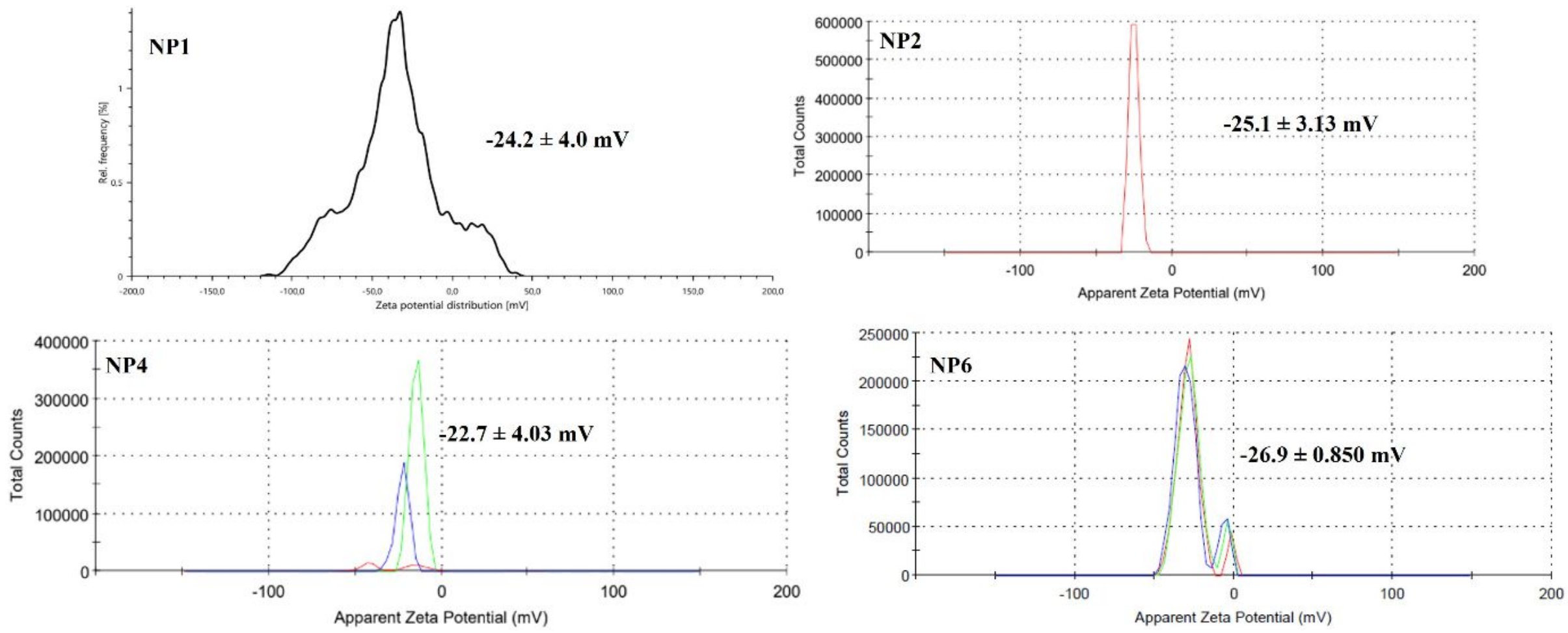

Fig. S5 Zeta potential measurements of non-targeted NP1 (non-coated NPs), NP2 (PLGA NP), NP4 (α-13′-COOH PLGA NP), and NP6 (GA-PLGA NP).

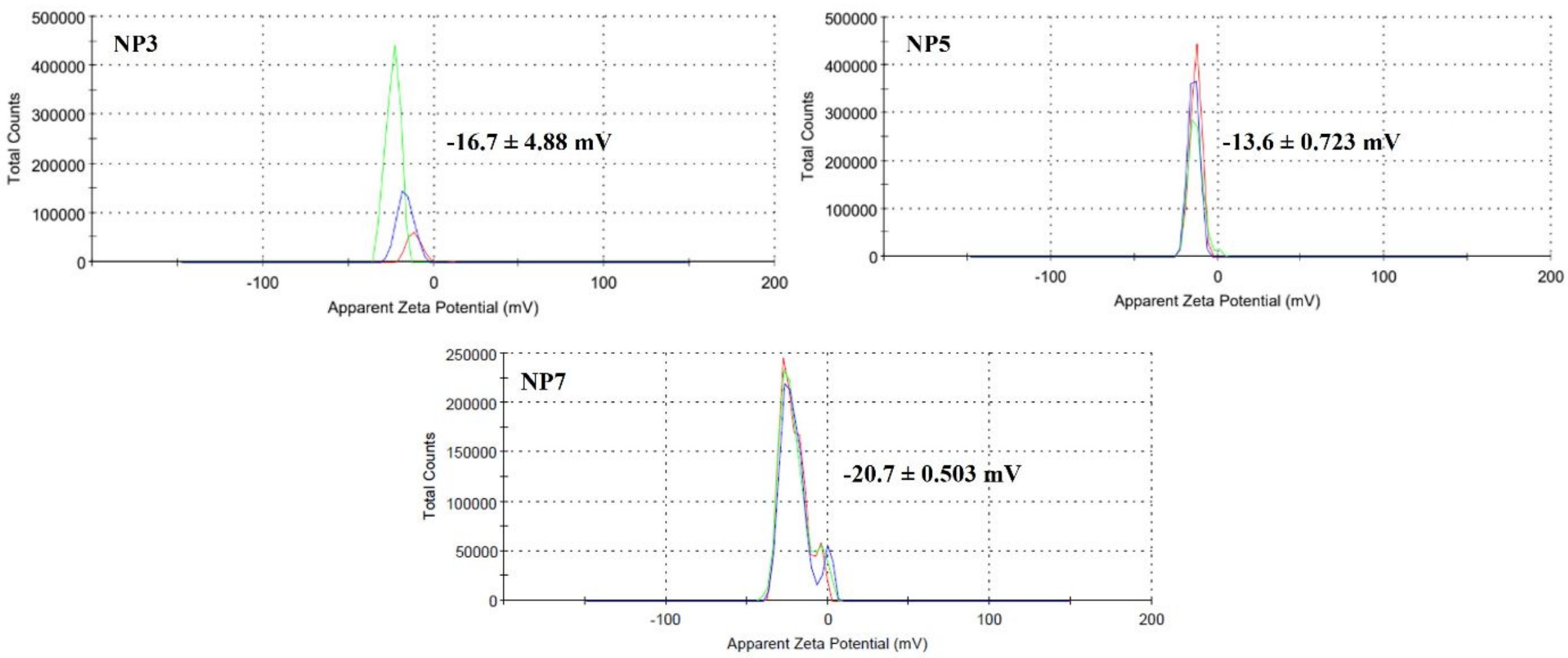

Fig. S6 Zeta potential measurements of targeted NP3 (S2P-PLGA NP), NP5 (S2P-α-13′-COOH PLGA NP), and NP7 (S2P-GA-PLGA NP).

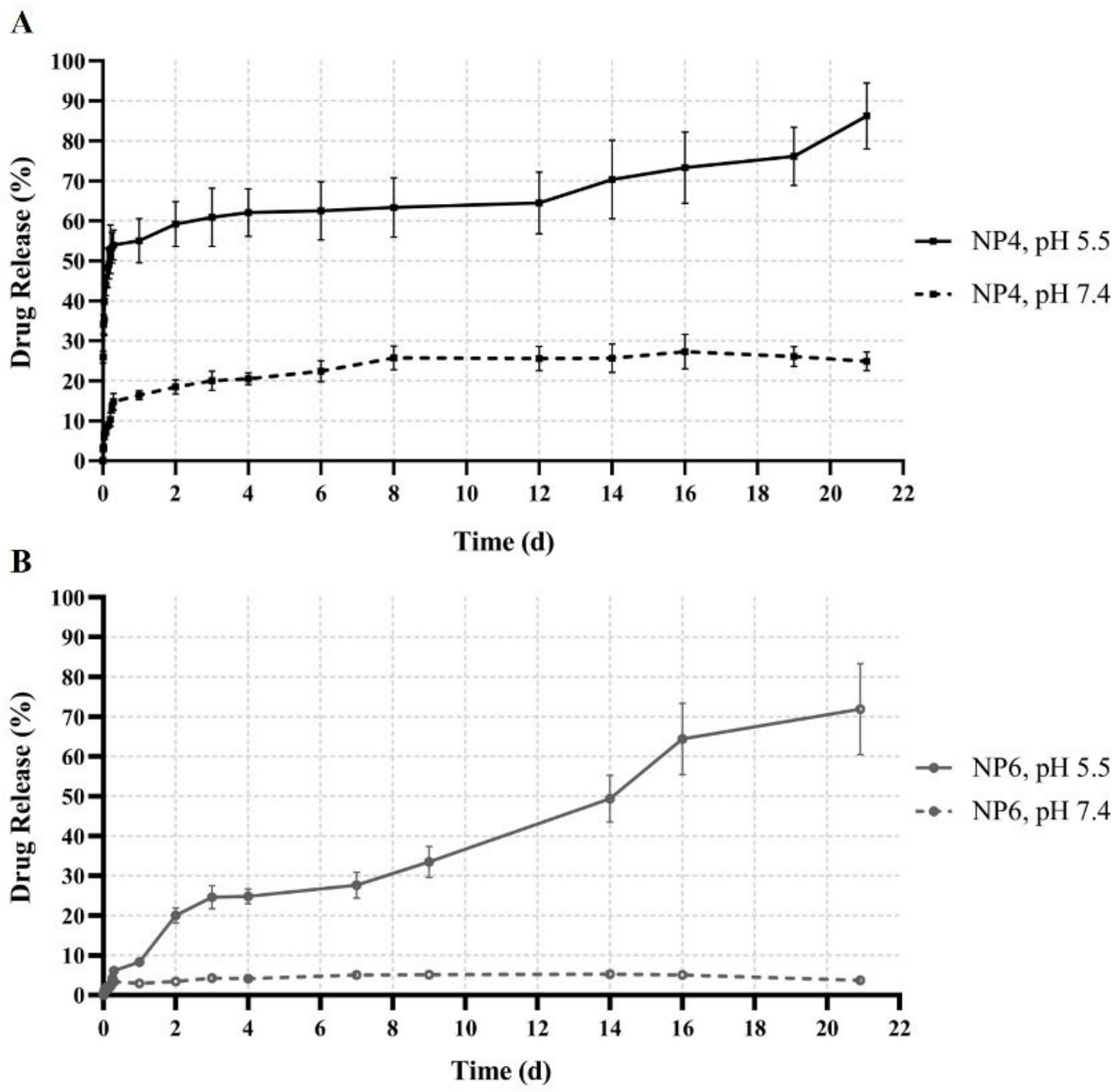

Fig. S7 Primary drug release profiles of NP4 and NP6 studied over 22 days. Data are presented as mean ± SD (n = 3).

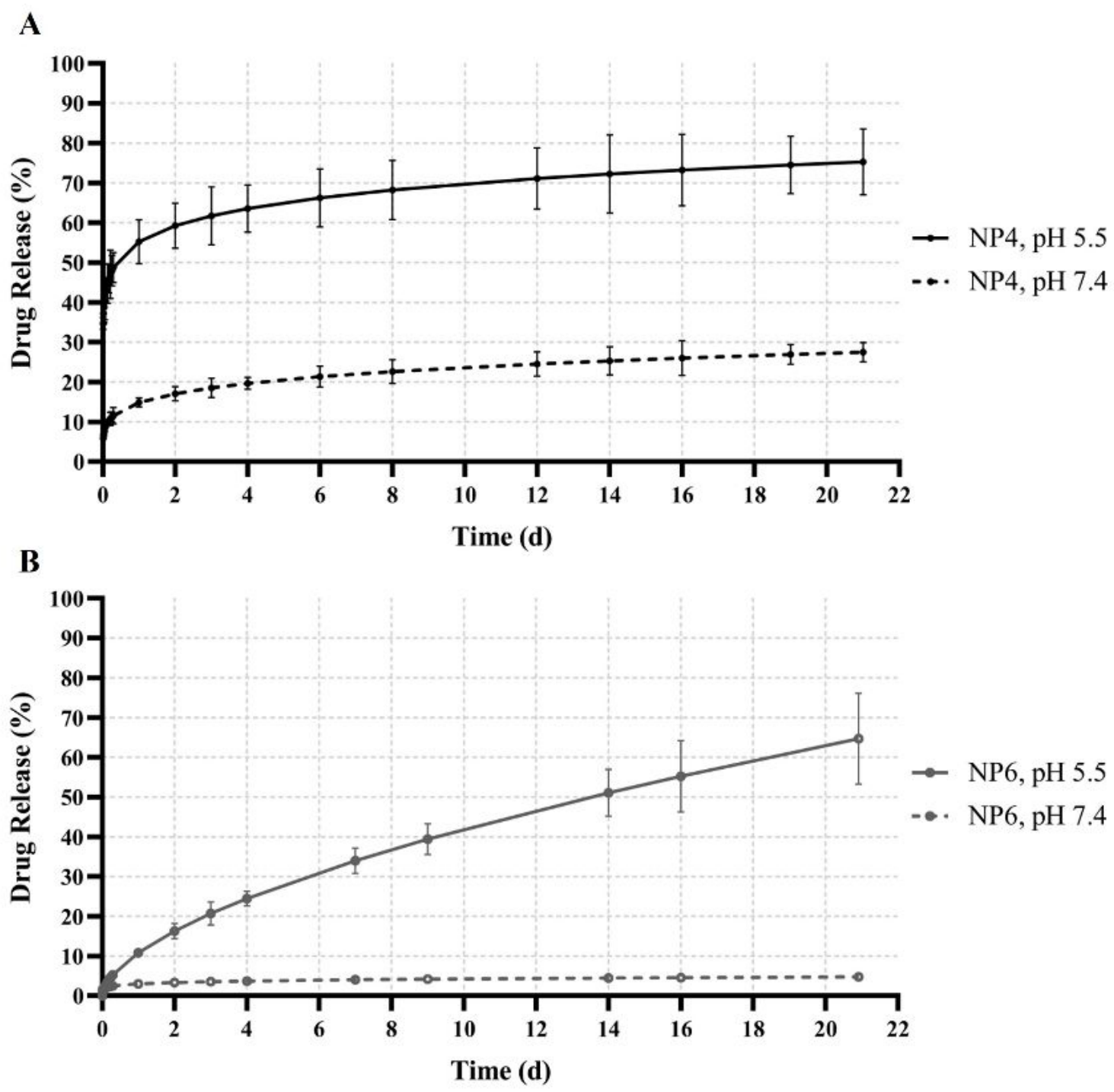

Fig. S8 Drug release profiles of NP4 and NP6 over 22 days, fitted using the Korsmeyer-Peppas model. Data are presented as mean ± SD (n = 3).

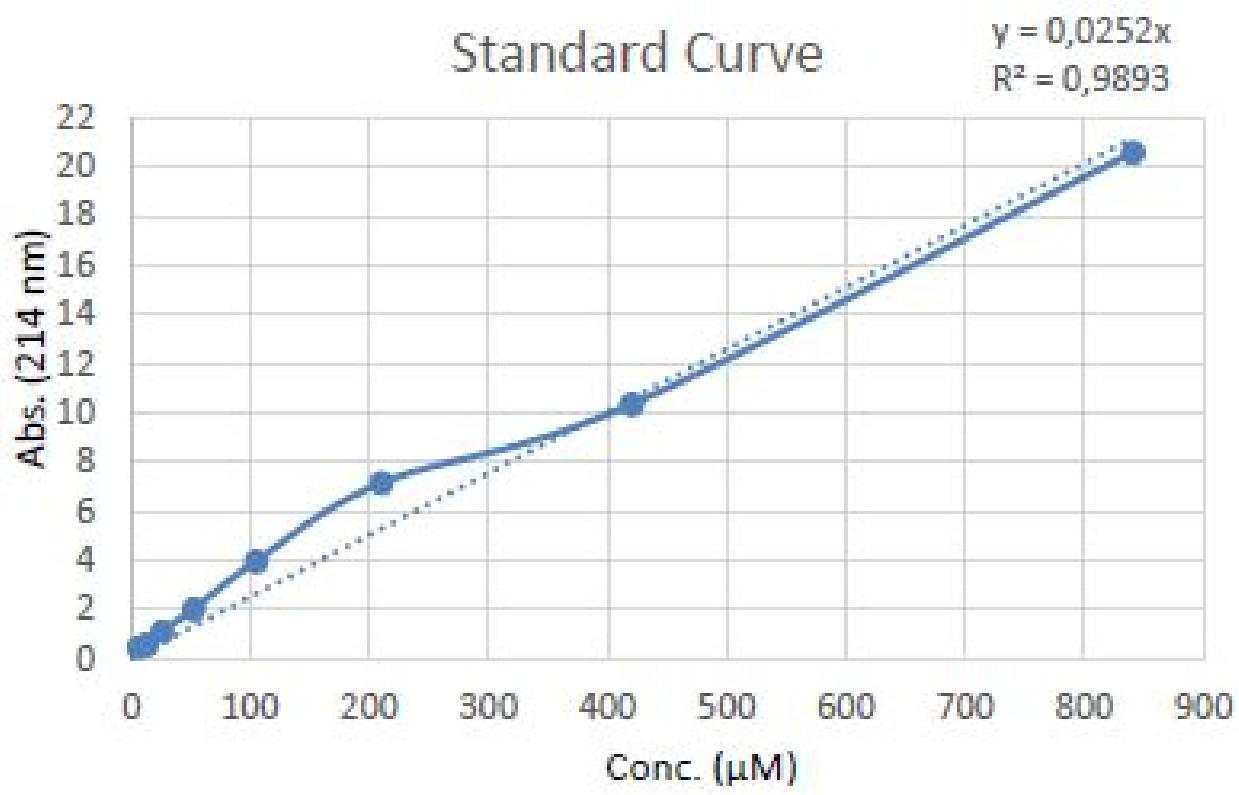

Fig. S9 Standard calibration curve used for S2P peptide quantification.
